# Cerebral Autoregulation in Subarachnoid Hemorrhage

**DOI:** 10.3389/fneur.2021.688362

**Published:** 2021-07-23

**Authors:** Darcy Lidington, Hoyee Wan, Steffen-Sebastian Bolz

**Affiliations:** ^1^Department of Physiology, University of Toronto, Toronto, ON, Canada; ^2^Toronto Centre for Microvascular Medicine at the Ted Rogers Centre for Heart Research Translational Biology and Engineering Program, University of Toronto, Toronto, ON, Canada; ^3^Heart & Stroke/Richard Lewar Centre of Excellence for Cardiovascular Research, University of Toronto, Toronto, ON, Canada

**Keywords:** stroke, microvascular dysfunction, cerebral blood flow, cystic fibrosis transmembrane conductance regulator, delayed ischemia

## Abstract

Subarachnoid hemorrhage (SAH) is a devastating stroke subtype with a high rate of mortality and morbidity. The poor clinical outcome can be attributed to the biphasic course of the disease: even if the patient survives the initial bleeding emergency, delayed cerebral ischemia (DCI) frequently follows within 2 weeks time and levies additional serious brain injury. Current therapeutic interventions do not specifically target the microvascular dysfunction underlying the ischemic event and as a consequence, provide only modest improvement in clinical outcome. SAH perturbs an extensive number of microvascular processes, including the “automated” control of cerebral perfusion, termed “*cerebral autoregulation*.” Recent evidence suggests that disrupted cerebral autoregulation is an important aspect of SAH-induced brain injury. This review presents the key clinical aspects of cerebral autoregulation and its disruption in SAH: it provides a mechanistic overview of cerebral autoregulation, describes current clinical methods for measuring autoregulation in SAH patients and reviews current and emerging therapeutic options for SAH patients. Recent advancements should fuel optimism that microvascular dysfunction and cerebral autoregulation can be rectified in SAH patients.

## Introduction

Cerebral aneurysms are common [1–5% prevalence (1, 2)] and pose a “silent risk” of severe brain injury. When an aneurysm ruptures, blood rapidly enters into the subarachnoid space: this event is termed aneurysmal subarachnoid hemorrhage (SAH) (1, 3, 4). In severe cases, intracranial pressure (ICP) elevates to levels that cause cerebrovascular arrest and death quickly ensues. As one might expect, SAH has a high case fatality rate (32–67%); of those that survive the initial bleed, 30-50% will suffer long-term disability as a result of serious brain injury (3–5). In terms of productive life years lost, SAH closely rivals more common forms of stroke due to its early age of onset (1, 6): thus, SAH incurs a disproportionately heavy cost (7), despite being a relatively rare form of stroke (~10 in 100,000 persons per year) (8–10). The interventions to halt ruptured aneurysm bleeding and prevent subsequent re-bleeds are frequently successful: thus, if the patient survives the initial bleeding event, which depends on the severity of bleeding and how quickly emergency medical attention is initiated, most of the *treatable* mortality and morbidity in SAH occurs during neurointensive care. In this regard, a pronounced secondary ischemic event, termed “*delayed cerebral ischemia (DCI)*” emerges 3–14 days following SAH. DCI is a significant cause of death and disability in SAH patients who survive the initial aneurysm rupture (1, 11, 12).

Until recently, DCI was attributed to radiographically visible large artery constriction, known as *angiographic vasospasm*, as this common complication often occurs concomitantly with the ischemic event (13). Consequently, the majority of research efforts focused on developing therapeutic interventions to curtail angiographic vasospasm, in the hopes that this would significantly improve patient outcome (4, 14). These efforts culminated in the disappointing CONSCIOUS clinical trials involving the endothelin-1 receptor antagonist clazosentan, which successfully reduced the incidence of large artery constriction, but failed to improve clinical outcome (15–18). This failure necessitated a shift in attention from the radiographically visible angiographic vasospasm to the radiographically invisible cerebral microcirculation. Indeed, given that the microcirculation is the primary determinant of cerebrovascular resistance (19), microcirculatory dysfunction is more aptly positioned to drive ischemic injury than large artery vasoconstriction. There are an extensive number of processes governed by the microcirculation; of these processes, the “automated” control of cerebral perfusion, termed “*cerebral autoregulation*,” appears to be an important aspect of SAH-induced brain injury, as it is clearly impaired following SAH (20–24) and it is a strong independent predictor of both DCI and negative outcome (22–24).

For physicians caring for SAH patients, this review summarizes the key clinical aspects of cerebral autoregulation and its disruption in the context of SAH. Our review is segmented into three primary subsections: (1) an overview of cerebral autoregulation, its mechanistic basis and predictions on how SAH alters autoregulatory function, (2) clinical measures of autoregulation and their relationship to patient outcomes, and (3) current therapeutic interventions for SAH in the context of autoregulation, which explains why alternative approaches are desperately required. In our subsequent discussion, we will examine some emerging therapeutic options that may be capable of correcting dysfunctional autoregulation in SAH.

### Cerebral Blood Flow Autoregulation

C*erebral blood flow autoregulation* is a regulatory mechanism that maintains constant brain perfusion over a relatively wide range of cerebral perfusion pressures (CPPs). This mechanism originates within the cerebral microcirculation, where resistance arteries actively match their level of constriction and hence, vascular resistance, to the prevalent perfusion pressure (25). Neils Lassen is credited with introducing the concept of cerebral blood flow autoregulation in 1959, after reviewing and integrating several human studies examining the effects of controlled hypotension on cerebral perfusion (26). However, the concept's roots date much further back, as the underlying myogenic mechanism had been identified by Sir William Bayliss in 1902 (27) and autoregulation had already been established within the renal microcirculation as early as 1946 (28).

**Figure 1:**
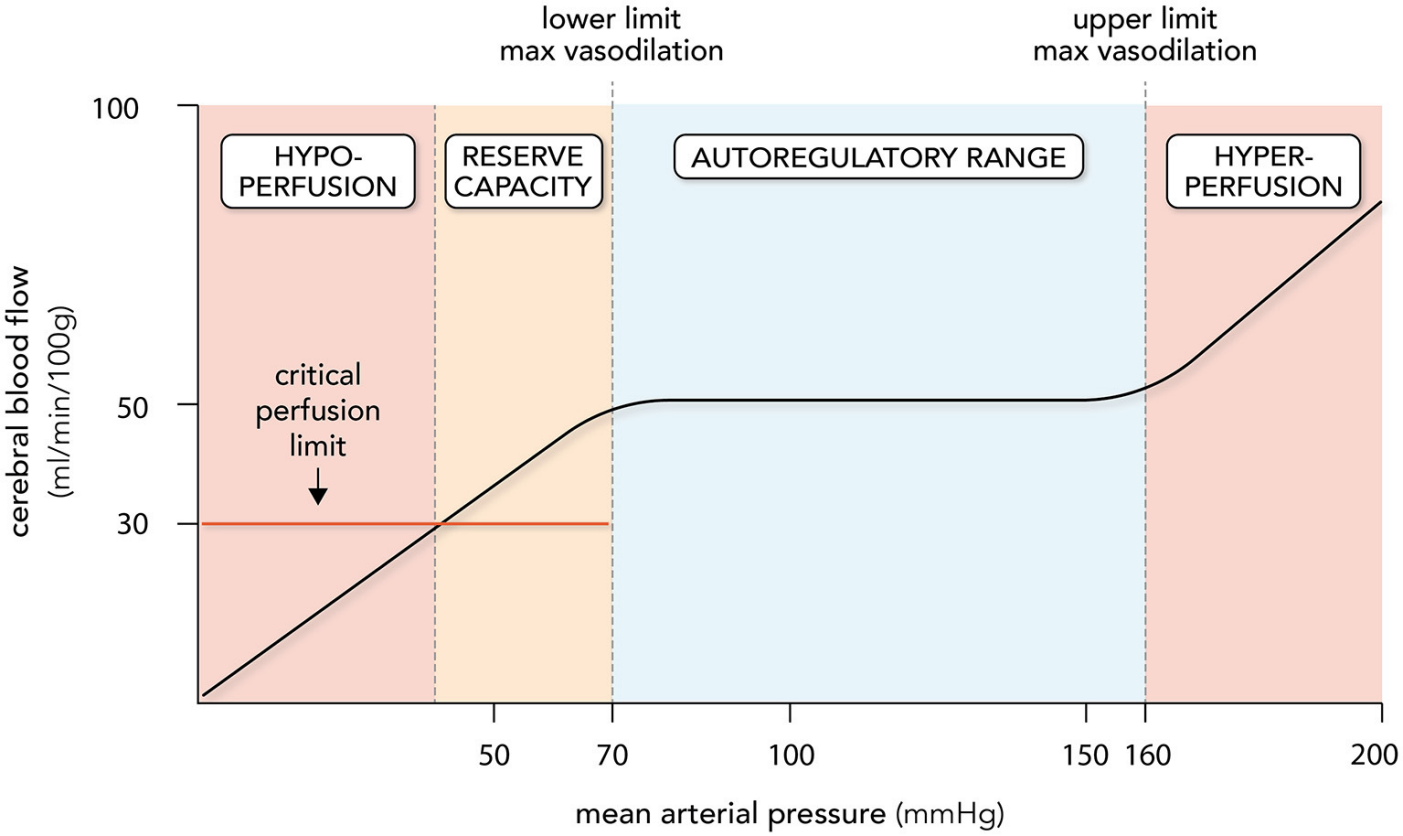
Cerebral autoregulation. Cerebral autoregulation is plotted as a relationship between cerebral blood flow (CBF) and mean arterial pressure (MAP). The autoregulatory range is defined by MAP levels that elicit maximal myogenic vasodilation (lower limit; ~70 mmHg) and myogenic vasoconstriction (upper limit; ~160 mmHg): CBF remains relatively stable as MAP changes within this range. Perfusion decreases when MAP drops below the lower limit; however, overt symptoms are not observed until a critical perfusion threshold is reached, usually 40–60% below normal levels. The MAP range where perfusion drops without symptoms is termed the cerebrovascular reserve capacity. Hypoperfusion and ischemia occur at MAP levels below the reserve capacity; hyperperfusion and vasogenic edema occur at MAP levels above the upper limit. It must be stressed that the lower and upper limits of autoregulation, the size of the cerebrovascular reserve capacity and the level of perfusion maintained by autoregulation all display variation. Thus, the plot represents regularly quoted values.

Lassen's curve visually presents cerebral autoregulation as a correlation between cerebral blood flow against mean arterial pressure (MAP; Figure 1) (26). Technically, CPP (i.e., the difference between MAP and intracranial pressure) is the more appropriate x-axis ordinate, but there is generally a tight relationship between MAP and CPP in normal settings, since ICP is generally constant. The autoregulatory curve possesses three key regions: a central plateau flanked by two inflection points that define the lower and upper limits of autoregulatory activity (Figure 1). The region at and below the lower limit is a state of maximal dilation within the microcirculation and consequently, reductions in CPP within this region result in reductions in blood flow. While the lower limit of autoregulation is considered a critical clinical benchmark, the brain is generally able to tolerate blood flow reductions of 30–60% before the onset of ischemic symptoms (29, 30). In essence, the brain enjoys a degree of “luxury perfusion,” known clinically as the “cerebrovascular reserve capacity.” Thus, the MAP at which ischemic symptoms typically arise may be substantially lower than the MAP that defines the lower limit of autoregulation. As an important caveat, SAH patients may have reduced or exhausted cerebrovascular reserve capacity (31) and thus, are likely to be more vulnerable to hypotension than healthy individuals. On the other side of Lassen's curve, the region at and above the upper limit of autoregulation represents a state of maximal microcirculatory constriction and at high pressures, an inability to maintain constriction (i.e., “forced dilation”). This poses a different threat to the brain's viability, including pressure-induced microcirculatory damage (32, 33), blood brain barrier disruption (34–36), and vasogenic edema formation (36, 37). Since blood brain barrier disruption and cerebrovascular edema are predictive for poor neurological outcome in SAH (38, 39), the upper limit of autoregulation is as clinically significant as the lower limit.

Since there are no systematic assessments defining the entire autoregulatory range in humans, the generally accepted range is 50-150 mmHg, as originally described by Lassen (25, 26). However, there are several key aspects of Lassen's work and our current understanding of autoregulation that are frequently overlooked (40). First, Lassen combined 11 different subject groups across 7 independent studies, regardless of patient health status or medication/anesthetic use: thus, the curve is highly prone to error, as it is composed of inter-subject means from uncontrolled and heterogenous conditions (26). As reviewed by Drummond (40), several subsequent studies specifically targeting the lower limit of autoregulation in humans suggest that the lower limit is actually much closer to a MAP of 70 mmHg. Second, the same studies reviewed by Drummond demonstrate remarkable inter-subject variability and consequently, the “one-size-fits-all” representation of the autoregulatory curve is considerably misleading: no single range can be broadly applied to a given patient (40). Third, the plateau of autoregulation is likely not perfectly flat, as there is evidence that autoregulatory responses to MAP elevation are more effective than responses to MAP reductions (41–44): thus, some degree of pressure passivity may be normal in healthy individuals and is not necessarily indicative of dysfunction (45). Finally, autoregulation is a physiologically fragile mechanism that is perturbed by many pathologies: thus, the autoregulatory range may be very different in SAH patients compared to healthy subjects and Lassen's curve (46–48). In summary, while the fundamental concept of autoregulation has withstood the test of time, the absolute values of Lassen's curve are outdated, and additional data derived from SAH patients is required in order to provide more effective guidance.

It must also be emphasized that pressure autoregulation is not the sole determinant of cerebral perfusion: in addition to automatically adjusting microvascular resistance to pressure, vascular smooth muscle cells also sense and integrate a variety of metabolic signals, which superimpose on autoregulation to elicit regional perfusion changes in response to heightened local metabolic demand (49–52). Examples of these metabolic signals include, but are certainly not limited to, pH, O_2_ and CO_2_ tensions, lactate, adenosine, nitric oxide, potassium ions, and vasoactive neurotransmitters (e.g., dopamine and acetylcholine) (49–52). These localized influences are most often termed “*neurovascular coupling*” (49–52), although “*metabolic autoregulation*” (i.e., the matching of perfusion to metabolic demand) has also been used (53). To avoid confusion, the present review refers to pressure autoregulation when the singular term “autoregulation” is used.

In pathological settings, autoregulatory dysfunction undoubtedly incorporates both intrinsic changes to how smooth muscle cells sense and respond to pressure and their vasomotor responses to the metabolic signals emanating from the external environment. Isolating the effects of SAH on the intrinsic pressure-sensitive mechanisms can only be effectively achieved with an *in vitro* experimental system that directly characterizes a resistance artery's response to pressure in a controlled external environment where confounding metabolic and neural inputs are eliminated.

### The Myogenic Response

Sir William Bayliss introduced the concept that intravascular pressure significantly modulates vascular tone in 1902, publishing a set of elegant *in situ* and *in vitro* (i.e., excised arteries) experiments that demonstrated pressure-dependent vasoconstriction and vasodilation (27). Since Bayliss could not attribute these responses to neuronal (i.e., responses persisted despite severed nerves and artery excision) or metabolic (stable *in vitro* conditions) inputs, he deemed these responses to be *myogenic* in nature (i.e., “myocyte origin”) (27). Indeed, Bayliss noted that “*The muscular coat of the arteries reacts, like smooth muscle in other situations, to stretching force by contraction… it also reacts to diminution of tension by relaxation, shown, of course, only when in a state of tone*.” (27). Remarkably, Bayliss' discovery was largely ignored for 45 years (54), in part because another prominent physiologist, Gleb von Anrep, failed to reproduce Bayliss' results in similar experiments and instead attributed the constrictions and dilations to neuronal (adrenaline) and metabolic influences, respectively (55). Although several subsequent studies by others largely supported Bayliss' original conclusion (54), the mechanism did not rise to prominence until (i) blood flow autoregulation was shown to be due to a non-neural, pressure-dependent mechanism (56) and (ii) myogenic mechanisms were demonstrated to significantly alter vascular resistance *in vivo* (57, 58).

As a functional definition, the myogenic response is vasoconstriction in response to an increase in transmural pressure (i.e., the pressure across the vessel wall) and vasodilation in response to a decrease in transmural pressure. Small cerebral arteries (<300 μm in diameter) are myogenically active (19, 59–62) and given that these arteries account for ~80% of the cerebrovascular resistance (i.e., between the systemic circulation and the cerebral capillaries) (19), they are the prime determinants of cerebral perfusion. It must be highlighted, however, that large cerebral arteries (~ 1 mm or more) (62, 63), including middle cerebral, basilar and pial arteries (64, 65), are also myogenically active. This is not typically observed in other tissues: in skeletal muscle, for example, only the smaller resistance arteries are myogenically active, whereas larger arteries appear to have minimal myogenic reactivity and a limited role in autoregulation within that tissue (19, 66). This point highlights a key danger of generalizations, based on observations from other vascular beds. In the cerebral circulation, there is clearly a gradient in myogenic reactivity, with smaller vessels developing more myogenic tone than larger vessels (62). Larger arteries may engage their myogenic mechanisms predominantly at higher pressures (>120 mmHg) (59, 67), which could be a means of increasing autoregulatory range. Specifically, to prevent small resistance arteries from reaching their maximal myogenic vasoconstriction limit at high pressures, cerebrovascular resistance increases upstream to attenuate the downstream pressures, thereby permitting the small resistance arteries to retain a high degree of perfusion control (59, 67). As mentioned previously, regional differences in autoregulation exist, indicating that myogenic reactivity and segmental activity within different regions of the brain is not uniform (59). Thus, different regions of the brain are likely to respond differently to SAH and systemic interventions.

At the molecular level, the myogenic response is the conversion of a mechanical stimulus (i.e., wall tension) into an intracellular biochemical signal; however, reviews over the last 20 years attest to the enormous complexity and mechanistic diversity of myogenic mechanisms (32, 54, 68–75). It is also noteworthy that mechanistic variations across species, biological sex, developmental status, vascular bed, and artery branch order have been identified (74). With this in mind, our review will provide only a simplified and abbreviated overview of selected key mechanisms driving myogenic signaling.

Depolarization is the critical initiator of the myogenic response: vascular wall tension is sensed by mechanosensitive ion channels (76–78) and/or other mechanosensitive elements (79–81), eliciting depolarization, L-type calcium channel activation, and calcium influx (82). Other voltage and ion-sensitive channels are subsequently engaged by the depolarization and ion influx, for example voltage gated (K_v_) and calcium-activated large conductance (BK) potassium channels, which play important roles in modulating the depolarization response (83, 84). Elevated cytosolic calcium levels stimulate *calcium-dependent vasoconstriction*, a process that involves the calcium/calmodulin-dependent activation of myosin light chain kinase and subsequent phosphorylation of myosin light chain 20: this phosphorylation event activates actin-myosin filament interaction and gliding (71). While extracellular calcium influx is generally considered to be the key source of calcium driving calcium-dependent vasoconstriction, calcium released from intracellular stores also plays a key role in mediating the constriction response (85, 86). In addition, calcium-independent mechanisms work in concert to increase *calcium sensitization*, which amplifies calcium-dependent responses by inhibiting the phosphatase that directly antagonizes the activity of myosin light chain kinase (i.e., myosin light chain phosphatase) (69, 71). In a previous review, we detailed a number of signaling entities that have been demonstrated to enhance calcium sensitivity in the context of the myogenic response (75): notable examples include sphingosine-1-phosphate (S1P) signaling (86, 87), 20-hydroxyeicosatetraenoic acid (20-HETE) signaling (72, 88), Rho kinase (89, 90), and protein kinase C (91, 92).

Only a handful of studies have utilized cannulated resistance arteries *ex vivo* to investigate the effects of SAH on myogenic reactivity (Table 1). As demonstrated in mouse olfactory (87, 93) and middle cerebral (94), rat parenchymal (95, 96) and middle cerebral (97), canine basilar (98), and rabbit cerebellar and posterior cerebral resistance arteries (99, 100), these studies are unanimous in concluding that SAH augments cerebral resistance artery myogenic reactivity. Through the use of endothelial denudation (95), smooth muscle cell specific gene deletion (87) and studies on freshly isolated artery myocytes (96, 100), this pathological effect can be attributed to a change in smooth muscle cell function. One common thread appears to be an enhancement of calcium-dependent signaling, which was either directly shown (95, 96, 100) or can be inferred (87, 93), based on previous studies (86, 90). Calcium sensitization is also likely to be enhanced, since the S1P-dependent signals characterized by Yagi et al., are known to enhance calcium sensitivity (69, 74, 86, 90). At the molecular level, (i) changes in potassium channel expression (96, 100) augment pressure-dependent depolarization and calcium influx (95), while (ii) augmented S1P signaling (87) further mobilizes intracellular calcium stores and increases calcium sensitization (86, 90). In summary, SAH appears to broadly increase myogenic reactivity throughout the cerebral microcirculation: since the basis of cerebral autoregulation is the myogenic response, these studies imply that autoregulation must also change in response to SAH.

**Table 1:** Research studies utilizing pressure myography to assess the effect of experimental subarachnoid hemorrhage on myogenic reactivity.

**References**	**Citation**	**Species**	**Cerebral artery**	**Critical observations**
Yagi et al., 2015	(87)	Mouse	Olfactory	SAH augments myogenic reactivity and reduces CBF. Etanercept (TNF inhibitor) and JTE-013 (S1P2 receptor antagonist) normalize myogenic reactivity and reduce neurological injury in SAH; etanercept improves CBF in SAH.
Lidington et al., 2019	(93)	Mouse	Olfactory	SAH augments myogenic reactivity and reduces CBF. Lumacaftor (CFTR corrector therapeutic) normalizes myogenic reactivity, improves CBF and reduces neurological injury in SAH.
Deng et al., 2018	(94)	Mouse	Middle Cerebral	Hemolyzed blood reversibly augments myogenic tone *in vitro* and *in vivo*. Superoxide scavenging (Tempol) prevents myogenic tone augmentation *in vitro* and *in vivo*.
Nystoriak et al., 2011	(95)	Rat	Parenchymal	SAH augments pressure-dependent membrane depolarization, calcium influx and vasoconstriction.
Wellman and Koide, 2013	(96)	Rat	Parenchymal	SAH augments potassium currents and myogenic tone.
Gong et al., 2019	(97)	Rat	Middle Cerebral	SAH increases TRMP4 expression/activity, resulting in augmented depolarization and vascular tone. Blockade of TRMP4 (9-phenanthrol) significantly improves vascular tone and CBF in SAH.
Harder et al., 1987	(98)	Dog	Basilar	SAH reduces basilar artery potassium conductance and induces basilar artery vasospasm. Inhibiting potassium efflux (nicorandil) reduces basilar artery constriction *in vitro* and i*n vivo*.
Ishiguro et al., 2002	(99)	Rabbit	Basilar, Posterior and Cerebellar	SAH augments posterior and cerebellar artery myogenic tone. SAH augments basilar artery constriction *in vivo* (measured angiographically).
Koide et al., 2011	(100)	Rabbit	Posterior and Cerebellar	SAH augments arterial wall depolarization, calcium influx and myogenic tone. SAH reduces calcium spark frequency, suggesting reduced BK channel activity.

*A blood injection model of SAH was used in all publications. BK, calcium activated large conductance potassium channels; CBF, cerebral blood flow; S1P2, Sphingosine-1-phosphate receptor 2; SAH, Subarachnoid hemorrhage; TRMP4, Transient receptor potential melastatin-4*.

### Autoregulatory Changes in Response to Augmented Myogenic Reactivity

**Figure 2:**
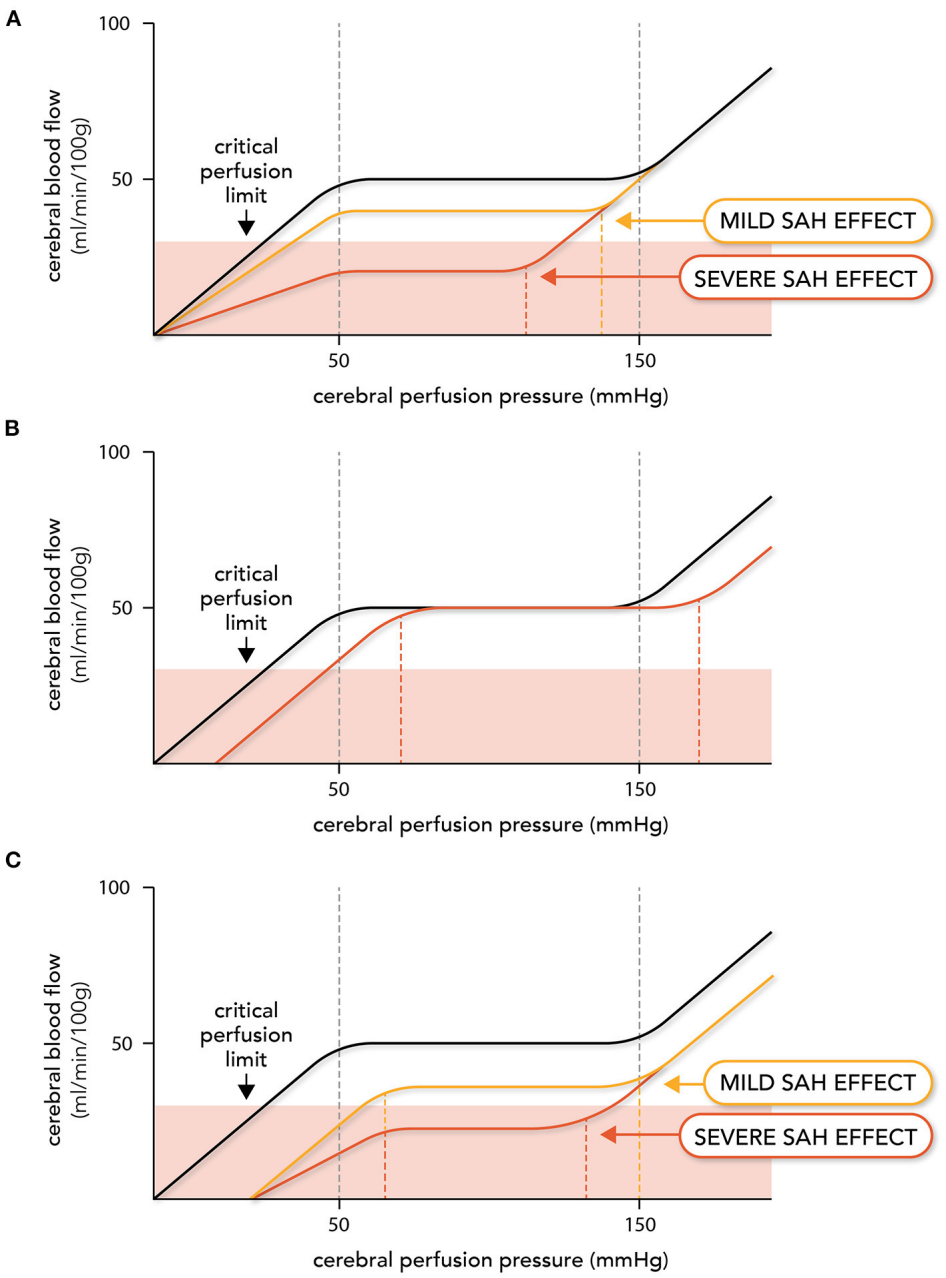
Expected effects of subarachnoid hemorrhage on autoregulation. Altered vascular reactivity in subarachnoid hemorrhage (SAH) can have three hypothetical effects on cerebral autoregulation. Note that in this figure, cerebral autoregulation is plotted as the relationship between cerebral blood flow (CBF) and cerebral perfusion pressure (i.e., the difference between mean arterial pressure and intracranial pressure): this was done because intracranial pressure varies in SAH patients, thereby adding a variable to consider when relating CBF to mean arterial pressure in this pathological setting. The autoregulation curve in black represents the normal, non-pathological situation, while the red and yellow lines represent altered autoregulation. In **(A)**, augmented myogenic reactivity (i.e., microvascular constriction) reduces perfusion, shifts the upper limit of autoregulation leftward and narrows the autoregulatory range. In severe cases, cerebral blood flow drops below the critical perfusion limit. In **(B)**, upstream large artery constriction (i.e., angiographic vasospasm) reduces the perfusion pressure entering the microcirculation. This stimulates a right-ward shift in the autoregulatory curve, but perfusion deficits do not occur until the new lower limit is reached. In **(C)**, both microvascular and larger artery constriction occur, creating a hybrid of **(A)** and **(B)**.

In clinical practice, it would be risky to assume that SAH does not alter the shape or position of the autoregulatory curve: thus, in order to properly interpret clinical measurements of autoregulation, we need to make predictions about how augmented myogenic vasoconstriction will affect autoregulation. Here, we will consider 3 potential scenarios involving microvascular and macrovascular constriction: (i) microcirculatory vasoconstriction (i.e., augmented myogenic vasoconstriction) in the absence of angiographic vasospasm, (ii) angiographic vasospasm alone, and (iii) both microcirculatory and large artery vasoconstriction. We propose the following effects on autoregulation:

Our first scenario (Figure 2A) is consistent with observations that DCI occurs in SAH patients in the absence of angiographic vasospasm (101–107). Augmented myogenic vasoconstriction reduces CBF and depending on the extent of the augmented myogenic reactivity, CBF may fall below the critical perfusion threshold, resulting in ischemia. The upper limit of autoregulation shifts to the left as a consequence of the enhanced constriction, assuming that the maximal constriction level for the artery is not altered. This is a reasonable conjecture, as the isolated artery studies found that SAH does not affect maximal artery diameter or enhance vasoconstriction to non-myogenic stimuli (87, 93, 95). One might speculate that the lower limit would also shift to the left, but there are two lines of evidence that suggest this would not occur. First, the pressure at which arteries lack wall tension (i.e., become slack) is very close to the lower limit of autoregulation: therefore, it is unlikely that mechanotransductive processes operate below the lower limit (108). Second, using hypocapnia as a means of inducing microvascular vasoconstriction and reducing CBF, Artru and Lam determined that the lower limit of autoregulation does not shift leftward as a result of enhanced vasoconstriction (109). Scenario 1 (Figure 2A) has been elegantly demonstrated in an experimental mouse model, where SAH induces (i) a marked reduction in CBF (ii) throughout a narrowed autoregulatory range that (iii) possesses a reduced upper limit and unchanged lower limit (110).

Because autoregulation maintains constant CBF at reduced levels, perfusion will not increase unless the CPP passes the upper limit of autoregulation. In severe cases (i.e., where ischemic symptoms are present), the autoregulatory range may be small enough that the upper limit can be passed with “moderate elevations” in systemic blood pressure [e.g., an elevation of mean arterial pressure to 140 mmHg (46); Figure 2A]. Since elevated blood pressure is common in SAH patients (111), some patients who do not have ischemic symptoms may have CPPs above the upper limit that would be identified as abnormal autoregulation.

In our second scenario (Figure 2B), large artery vasoconstriction (i.e., angiographic vasospasm) would increase upstream resistance, thereby lowering the intravascular pressure sensed by the microvascular resistance arteries. In response, the resistance arteries would dilate to maintain CBF, until the lower limit of autoregulation is reached. Thus, the primary effect large artery vasoconstriction is a rightward shift in the autoregulatory curve, but not a change in the perfusion level. In severe angiographic vasospasm, the autoregulatory curve may shift far enough that CPP falls below the lower limit and reductions in perfusion are observed; however, some patients will remain in the reserve capacity zone and therefore, not suffer ischemic injury as a result (112). This scenario is consistent with observations that many patients with severe angiographic vasospasm do not suffer significant neurological decline (14, 112). Scenario 2 (Figure 2B) has been demonstrated in an experimental rabbit model associated with significant vasospasm: in this model, SAH profoundly compromises the lower limit of autoregulation, with CBF eventually reaching control levels at higher mean arterial pressures (113).

Our final scenario (Figure 2C) combines both of these elements: cerebral perfusion is reduced; the autoregulatory range narrows due to a leftward shift in the upper limit; and the autoregulatory curve shifts to the right due to the large artery constriction decreasing the “apparent” intravascular pressure sensed by the microcirculation. This represents a potentially dangerous scenario for SAH patients, as the rightward shift in the autoregulatory curve has the potential to shift the patient's CPP below the lower limit, thereby further reducing perfusion. Curtailing angiographic vasospasm may or may not increase cerebral perfusion in this scenario; even if perfusion increases, it may not sufficiently rise to prevent ischemic injury. This scenario provides a possible explanation for why therapeutically targeting angiographic vasospasm failed to deliver benefit (15, 16).

## Clinical Data on Cerebral Autoregulation

Cerebral autoregulation has been a focus of clinical assessment in SAH patients for over 50 years. In this subsection, we present distilled descriptions of the most frequently used methods to measure autoregulation in SAH patients, including the key principles, caveats, and conclusions.

### Static Autoregulation Measurement

Direct cerebral blood flow measurements require lengthy time scales and consequently, provide only a snapshot of autoregulation in steady state settings (114). Fantini et al. provide a comprehensive review of the techniques utilized to quantitatively measure cerebral blood flow, which notably includes ^133^Xe clearance, positron emission tomography, thermal diffusion, magnetic resonance imaging arterial spin labeling, and perfusion computed tomography (114). Utilizing these approaches for autoregulation studies generally requires pharmacological interventions to persistently alter blood pressure, although positional changes can also be used. Transcranial Doppler (TCD) has been extensively used to assess static autoregulation in shorter time periods (41), but measuring blood flow velocity (BFV) as a surrogate for blood flow is error-prone, due to the poor assumption that the insonified artery's diameter does not change in response to the pharmacological intervention (113, 115, 116). The emergence of color-coded duplex ultrasonography (CDUS), which simultaneously measures BFV and arterial diameter, provides a superior ultrasound alternative to the standard TCD approach (115).

Static autoregulation measurements generally provide quantitative measures of the overall performance of autoregulation, in many cases with a high spatial resolution. In fact, Lassen's iconic autoregulatory curve was the result of combining 7 human studies with static measurements of cerebral blood flow and MAP (26). However, the approach is not without limitations, especially in SAH patients: (i) pharmacologically inducing blood pressure changes can be dangerous in critically ill SAH patients, (ii) sequential monitoring can require lengthy periods of time and therefore, it is not practical to routinely monitor static autoregulation, (iii) certain methods, such as ^133^Xe clearance, are invasive (intra-arterial injection) (114), (iv) anesthetics (e.g., halothane) and certain vasoactive agents very likely alter autoregulation, thereby incorporating a potential confound and (v) most measurements require non-bedside equipment.

Although autoregulation responds to both increases (vasoconstriction) and reductions (vasodilation) in perfusion pressure, running both protocols in SAH patients is logistically challenging. Thus, studies generally measure responses after a single stimulus in SAH patients, almost always a reduction in blood pressure (20, 101, 117–121). In some cases, a hypertensive stimulus has been utilized (122, 123) and in others no stimulus was utilized (124, 125). In this latter case, autoregulatory failure is inferred when angiographic vasospasm reduces downstream perfusion, due to an assumption that compensatory downstream vasodilation should engage (124). It is worth noting that the majority of static autoregulation studies are rather dated and that the standard of care for SAH patients has evolved considerably since.

Given that ischemia and perfusion deficits are common in SAH patients, it is not surprising that these studies largely confirm that SAH compromises autoregulation in many SAH patients (Table 2) (20, 101, 117–125). There is little and disparate data regarding the temporal profile of autoregulatory perturbation, with one study indicating that the initial phase displays the greatest autoregulatory disruption (<7 days) (20), while another suggests that significant deterioration occurs within the window of delayed ischemia (>7 days) (121). Similarly, there is little data relating these measures to outcome, with one study indicating that highly-disrupted autoregulation associates with poor Glasgow outcome score (117), while another found no autoregulatory differences between patients who develop symptomatic vasospasm vs. those who do not (121). Intriguingly, two studies indicate that autoregulatory dysfunction is heterogeneous and consequently, systemic interventions intended to increase blood flow within ischemic regions can paradoxically reduce perfusion in that region and/or others (101, 122). This raises concerns that empirically deploying systemic therapies may have serious unintended consequences.

**Table 2:** Clinical studies assessing cerebral blood flow autoregulation in subarachnoid hemorrhage patients.

**References**	**Citation**	**Study size**	**SAH grade**	**CBF method**	**Critical observations**
Dernbach et al., 1988	(20)	SAH - 14/Unruptured - 10	Hunt-Hess 1-4	Thermal Probe	Patients with Hunt-Hess 1-2 scores display perturbed autoregulation when operated on within 7 days of SAH (*n* = 6). After 7 days post-SAH ictus (*n* = 8), perturbed autoregulation was not evident.
Heilbrun et al., 1972	(101)	SAH - 10	Hunt-Hess 2-5	Intra-arterial ^133^Xe CT	4/9 SAH patients displayed a global loss of autoregulation. 4/5 patients with intact autoregulation had satisfactory outcomes, while 3/4 patients with disrupted autoregulation had poor outcomes.
Tenjin et al., 1988	(117)	SAH - 9/Unruptured - 3	WFNS 1-4	Thermal Probe	SAH severity predicts perturbed autoregulation (*n* = 2 WFNS 3-4 vs. *n* = 7 WFNS 1-2); perturbed autoregulation predicts outcome (*n* = 4 GOS 3-5 vs. *n* = 5 GOS 1-2).
Muench et al., 2005	(118)	SAH - 10	Hunt-Hess 2-5	TD-rCBF	Pathological values of autoregulation index were observed in the patient population. Reductions in mean arterial pressure result in decreased cerebral blood flow.
Nornes et al., 1977	(119)	SAH - 21	Hunt-Hess 1-3	Flow Probe	Grade 3 SAH (*n* = 9) induces a rightward shift in the lower limit of autoregulation (~76 mmHg), compared to grades 1-2 (*n* = 12; ~62 mmHg).
Pickard et al., 1980	(120)	SAH - 20	Hunt-Hess 1-3	Intra-venous ^133^Xe CT	Halothane-induced hypotension increased CBF in 15/20 patients, with 1 developing neurological deficits; 5/20 had reduced CBF during hypotension, with 4 developing neurological deficits.
Cossu et al., 1999	(121)	SAH - 77	WFNS 1-5	Thermal Probe	WFNS grade 1-2 patients (*n* = 23) had a better autoregulatory index than WFNS 4-5 patients (*n* = 19). In WFNS grade 1-2 patients, better autoregulation was observed within 0–2 days post-SAH compared to 3–7 days and >7 days.
Darby et al., 1994	(122)	SAH - 13	Hunt-Hess 1-5	Inhaled ^133^Xe CT	Dopamine-induced hypertension (90–110 mmHg) does not alter overall CBF; however, ischemic territories increase CBF while high perfusion territories decrease CBF.
Muizelaar et al., 1986	(123)	4 SAH Case Reports	Hunt-Hess 2-5	^133^Xe CT	Phenylephrine-induced hypertension (17–50 mmHg) increases CBF. 3/4 patient MAPs were within the normal autoregulatory range prior to (90–98 mmHg) and following intervention (112–126 mmHg).
Hattingen et al., 2008	(124)	SAH - 51/Healthy - 15	Hunt-Hess 1-5	MRI Spin Labeling	SAH patients have reduced CBF compared to controls. Vasospasm reduces CBF in downstream region. Impaired autoregulation is inferred by lack of compensatory vasodilation, as measured by CBV.
Diringer et al., 2016	(125)	SAH - 25	WFNS 2-5	^15^O PET	Normal mean autoregulatory index following phenylephrine treatment. However, data values were highly variable, with many points outside “normal” range.

*CBF, cerebral blood flow; CBV, cerebral blood volume; CT, computed tomography; GOS, Glasgow outcome score; MAP, mean arterial pressure; MRI, magnetic resonance imaging; PET, positron emission tomography; SAH, Subarachnoid hemorrhage; TD-rCBF, Transcranial Doppler relative CBF measurement; WFNS, World Federation of Neurosurgical Societies scale*.

### Dynamic Autoregulation Measurement

Dynamic autoregulation assessment refers to a relatively simple TCD-based method that evaluates the autoregulatory response to a transient reduction in perfusion pressure. In brief, middle cerebral artery (MCA) BFV is continuously measured by TCD. Autoregulatory responses are then measured following an alteration in CPP (the transmural pressure sensed by myogenic mechanisms): this is typically achieved in SAH patients by compressing the carotid artery ipsilateral to the insonified MCA (126–131), although in some cases, a bilateral thigh cuff is utilized (132, 133). Compression of the carotid artery (tailored to yield ~30% reduction in MCA BFV) decreases CPP: after a brief period (usually 3–5 s), the compression is released. When autoregulation is behaving normally, the microcirculation dilates in response to the reduced perfusion pressure: consequently, when pressure is rapidly restored, a brief hyperemic response occurs. Hence, the test is frequently referred to as a ***Transient Hyperemic Response Test (THRT)***. The test is scored as a binary measure (intact/perturbed), depending on whether the transient hyperemic BFV is 10% higher than the pre-compression BFV. In the thigh cuff approach, thigh cuffs are inflated to 200 mmHg for 2 min and then rapidly released, resulting in a negative step change in CPP. The autoregulatory BFV response is then mapped to 10 theoretical traces incorporating autoregulatory dynamic gain, a dampening factor and a time constant (134). Each curve represents a different degree of autoregulatory impairment, known as the ***Autoregulation Index (ARI)***, with 0 indicating no autoregulation and 9 indicating “perfect” autoregulation.

Although the methods are relatively simple, non-invasive and use readily available TCD equipment, there are some general caveats and limitations: (i) the measurements are highly prone to inconsistency, most likely due to short-term variations in autoregulatory activity, the influence of other physiological variables and noise in the system, (ii) there is an assumption that MCA diameter does not change as a result of the interventions, and (iii) the THRT is graded against a somewhat arbitrary present/absent threshold that oversimplifies the outcome.

SAH patients frequently display abnormal THRT results (Table 3) (126–131). Two studies show a timeline following SAH ictus, based on the proportion of patients with negative/abnormal THRT results: while these studies show variable proportions of negative results early after ictus (0–3 days post-SAH), there is a clear peak between days 7–10, after which point the proportion of negative THRT results declines (128, 129). This peak fits well with the timeline for development of DCI. Negative THRT results are frequently, but not always, a good predictor for the emergence of angiographic vasospasm (126, 127, 129, 130) and poor THRT results are clearly more prevalent in patients who develop cerebral infarction (129, 131), have poor neurological status (e.g., WFNS Score >2 or Glasgow Coma Score <6) (126–129, 131) and ultimately have poor outcomes (e.g., Glasgow Outcome Score ≤3 or modified Rankin Scale ≥4 at 6 months) (126, 128, 131). Likewise, the thigh cuff/ARI approach appears capable of segregating patients who will develop angiographic vasospasm, rapid neurological deterioration, and suffer poor outcome (132, 133). ARI is consistently reduced in SAH patients (ARI scores of 4–6 in good outcome and 1–4 in poor outcome) and generally increase over time (i.e., over 0-10 days post-SAH) in patients who improve and decline in those who deteriorate (132, 133).

**Table 3:** Clinical studies utilizing the Transient Hyperemic Response Test (THRT) in subarachnoid hemorrhage patients.

**References**	**Citation**	**Study size**	**SAH grade**	**Critical observations**
Smielewski et al., 1995	(126)	52	WFNS 1-5	Negative THRT result (<1.09) correlated with worse WFNS grade and GOS.
Lam et al., 2000	(127)	20	WFNS 1-4	6/20 patients displayed a negative THRT result (<1.09) 1 day after surgery: 5 developed DIDs.6/14 remaining patients had a negative THRT result 3–7 days post-surgery: none developed DIDs.
Rätsep and Asser, 2001	(128)	55	WFNS 1-5	A negative THRT result (<1.10) was found in 22–35% of assessments over 0–19 days post-SAH ictus.Negative THRT results peaked at 0–3 and 7–14 days. Negative THRT associated with unfavorable GOS (1-2).
Rätsep et al., 2002	(129)	50	WFNS 1-5	Negative THRT results (<1.10) were found in 33% of assessments over 0–18 days post-SAH ictus. Negative THRT results peaked at 0–3 and 7–14 days post-SAH. Negative THRT result associated with poor initial
				WFNS grade (>2), vasospasm and impaired consciousness.
Al-Jehani et al., 2018	(130)	15	Hunt-Hess 1-5	7/15 patients had a negative THRT result (<1.09). A negative THRT result predicts the development of symptomatic vasospasm (5/6).
Rynkowski et al., 2019	(131)	40	Not Defined	19/40 patients had a negative THRT result (<1.09). Negative THRT result correlated with Hunt-Hess score ≥4, higher APACHE II scores (12 vs. 3.5) and unfavorable outcome (mRS ≥4 at 6 months).

*These studies included SAH patients only. In Rynkowski et al., Hunt-Hess scores were obtained for 11/40 patients. APACHE II, Acute Physiology And Chronic Health Evaluation II; DIDs, Delayed ischemic deficits; GOS, Glasgow outcome score; mRS, Modified Rankin score; SAH, Subarachnoid hemorrhage; THRT, Transient hyperemic response test; WFNS, World Federation of Neurosurgical Societies scale*.

### Continuous Autoregulation Measurement

There are several practical considerations that hinder the routine measurement of static and dynamic cerebral autoregulation in critically ill patients: (i) static autoregulation measures require the use of vasoactive drugs or mechanical maneuvers that are often contraindicated in critically ill patients, (ii) assessments can be time consuming and consequently, frequent/routine assessment would add a substantial workload burden to neurocritical care staff, and (iii) assessments can be prohibitively expensive, especially if MRI-based methods are used. In a critical care setting, cerebral autoregulation assessments should be fast and inexpensive, minimally disturb the patient and ideally, utilize data that is already collected as part of neurocritical monitoring. Continuous autoregulation measurements largely meet these criteria, as they rely on endogenous variations in blood pressure and utilize continuous monitoring data from widely available equipment.

The ***Pressure Reactivity Index (PRx)***, first described in 1997 by Czosnyka et al. (135, 136), measures the association between slow waves in arterial blood pressure (ABP) and ICP, where ABP serves as a surrogate for CPP and ICP as a surrogate for vascular reactivity. The latter is predicated on the fact that vasomotor responses globally change cerebrovascular volume and consequently, ICP (as per the Monro-Kellie doctrine). In a setting with intact autoregulation, there is an inverse relationship between ABP and ICP: increases in ABP stimulate myogenic vasoconstriction, which decreases ICP *via* a blood volume reduction. The PRx index is calculated using a moving Pearson correlation; when PRx is subsequently plotted against CPP (i.e., CPP=ABP-ICP), a U-shaped relationship is observed (137), with the autoregulatory range defined by near-zero or slightly negative values and the progressive loss of autoregulation as increasingly positive values (i.e., increased ICP resulting from the transmission of ABP into the microcirculation).

Although measuring PRx requires is invasive (i.e., ventricular or intraparenchymal transducer), these parameters may be measured in SAH patients who are subject to continuous ICP monitoring. However, PRx measurements in SAH patients possesses key caveats: (i) ABP must be a reliable correlate of CPP, which may be problematic in patients with high ICP levels, putatively due to compression of intracranial veins and consequent increase in venous resistance (138, 139); (ii) slow waves in ICP must be solely dependent on blood volume changes, as experimentally validated in non-pathological settings (140); (iii) craniospinal compliance (i.e., the capacity to compensate for added intracranial volume) must not substantially increase following decompressive craniotomy (141, 142) or with the use of external ventricular drains (143), as this will alter PRx values regardless of autoregulatory status [decompressive craniotomy likely invalidates PRx measurements (142)]; and (iv) as a global measure, PRx measurements assume that all vascular beds within the cerebral microcirculation behave similarly with respect to pressure reactivity, which is probably true at normal perfusion pressures, but not at the extremes (144). It should also be noted that since the CPP estimation incorporates the ICP (i.e., CPP=ABP-ICP), there is a statistical bias in the U-shaped PRx/CPP relationship, owing to the fact that PRx and CPP share a common parameter, resulting in a degree of “autocorrelation” (145). Without correction, this bias likely distorts the true autoregulatory window and consequently, the estimates of appropriate perfusion pressures (145).

PRx measurements are more firmly established as a prognostic indicator for traumatic brain injury patients than they are for SAH patients (136, 146). Nevertheless, there are several informative PRx studies in SAH patients (Table 4) (46, 143, 147–151). The vast majority of SAH patients display abnormally positive PRx values (i.e., perturbed cerebral autoregulation) (46, 143, 147–149); as anomalies, one small study (21 patients) failed to show disrupted autoregulation in SAH patients (151) and another (42 patients) found normal autoregulation in patients who survived for 3 months, while non-survivors displayed significantly higher PRx values (150). A subset of studies measured PRx over a prolonged time course (i.e., within 1 day to 14 days post-SAH) (46, 147, 149): as a general trend, PRx values were highly positive initially, declined between days 1–3 and then rebounded to higher levels over the next 7-10 days (46, 147). The acutely elevated PRx levels (days 0–3) may indicate (or contribute to) the extent of early brain injury (152), while the delayed rise in PRx on day 4 and beyond is consistent with the timeframe that DCI clinically emerges (11).

**Table 4:** Clinical studies utilizing Pressure Reactivity Index (PRx) measurements in subarachnoid hemorrhage patients.

**References**	**Citation**	**Study size**	**SAH grade**	**Critical observations**
Svedung Wettervik et al., 2021	(46)	242	WFNS 1-5	PRx was >0 in SAH patients and tended to increase at 3–4 days post-ictus in patients with unfavorable outcome (GOS-E 1-4 at 12 months). High PRx values independently associate with unfavorable outcome.
Howells et al., 2017	(143)	129	Hunt-Hess 1-5	80/129 patients had an extraventricular drain opened during ICP/PRx measurements. An open drain did not corrupt ICP signal and conferred small, but significant improvements in PRx.
Gaasch et al., 2018	(147)	43	Hunt-Hess 2-5	PRx values are highest at day 0 post-ictus (0.31), decline and then rise at 4–10 days post-ictus. Patients with DCI and poor outcome (mRS 3-5 at 3 months) had higher PRx values compared to those without. High PRx values over 0–3 days post-ictus (0.21 vs. 0.08) associated with DCI and poor outcome.
Johnson et al., 2016	(148)	47	Hunt-Hess 1-5	Patients with PRx >1 had lower CBF than PRx ≤1 patients over 14 day assessment period. Dichotomized PRx groups did not associate with Hunt-Hess score or predict the development of DCI.
Eide et al., 2012	(149)	94	Hunt-Hess 1-5	PRx was higher (0.28) in patients who die (mRS 6), compared to mRS 0-2 (0.16) and mRS 3-5 (0.12) patients. PRx could not differentiate mRS 0-2 and mRS 3-5 patients. Amplitude correlation was a better predictor than PRx.
Bijlenga et al., 2012	(150)	42	WFNS 4-5	PRx at 0–2 days post-ictus was higher in patients who died within 3 months (0.10; 9/25) vs. survivors (−0.17; 16/25). PRx did not predict the development of vasospasm; PRx values were not significantly affected by vasospasm.
Barth et al., 2010	(151)	21	Hunt-Hess 2-4	PRx values were not statistically different between patients who developed infarcts (0.06; 8/21) vs. those who did not develop infarcts (0.10; 13/15). PRx did not correlate with ORx or FRx indies.

*These studies included SAH patients only. With the exception of Eide et al., all studies were retrospective. DCI, delayed cerebral ischemia; FRx, flow reactivity index; GOS-E, Glasgow outcome score-extended; ICP, intracranial pressure; mRS, Modified Rankin score; ORx, oxygen reactivity index; PRx, Pressure reactivity index; SAH, Subarachnoid hemorrhage; WFNS, World Federation of Neurosurgical Societies scale*.

The prognostic value of PRx measurements in SAH patients is not yet established. While several studies observe an association between higher PRx values and outcome parameters (46, 147, 150), others have not (148, 149, 151). Small numbers, variations in SAH severity and therapeutic interventions, different threshold values for abnormal PRx, technical differences (e.g., probe placement) and PRx calculation differences [e.g., signal averaging (149)] likely contribute to these discrepancies. In a 242 patient retrospective cohort study (46), higher PRx levels at 6.5–10 days post-SAH independently associate with unfavorable extended Glasgow outcome scores (GOS-E ≥4 at 12 months); interestingly, CPP levels *below* 90 mmHg (normal CPP target is 60-70 mmHg) also associated with unfavorable outcome. This potentially identifies a clinically significant augmentation of myogenic vasoconstriction, which would simultaneously induce a leftward shift in the upper limit of autoregulation (resulting in higher PRx values) and reduce perfusion (promoting ischemia at CPP levels of 60-70 mmHg). In support of this conclusion, Johnson et al., stratified SAH patients into high and low PRx values (≤0.1 vs. >1) and found that the high PRx group associated with lower CBF, despite the fact that both groups possessed similar/normal CPP and ICP levels (148). Interestingly, neither of these studies found a significant relation between PRx or CBF and development of symptomatic DCI (46, 148).

Of the other continuous methods for determining cerebral autoregulation, it is worth elaborating on the TCD-based ***Systolic Reactivity Index (Sx)***and ***Mean Reactivity Index*** ***(Mx)***, as these are non-invasive alternatives to PRx. Both Sx and Mx are similar to PRx, in that they correlate slow waves in CPP and CBF surrogates. For Sx and Mx, systolic blood pressure (Sx) and mean blood pressure (Mx) serve as surrogates of CPP, while MCA BFV serves as a surrogate of CBF. In a setting with intact autoregulation, CBF remains constant despite changes in blood pressure and consequently, MCA BFV remains constant; when autoregulation is compromised, CBF increases with pressure and hence, BFV in the MCA concomitantly increases. Like PRx, the Sx and Mx indices are calculated using a moving Pearson correlation, yielding a U-shaped autoregulatory relationship.

Although measuring Sx or Mx is non-invasive, the method has several caveats and limitations: (i) the technique is highly operator-dependent and requires expertise for reliable and reproducible measurements (153); (ii) the measurement is applicable only to the territories perfused by the MCA; (iii) although TCD equipment is readily available in critical care settings, TCD monitoring in critically ill patients is cumbersome and generally time-limited to 1 h (138, 142); (iv) like PRx, Mx and Sx are impacted by high ICP, presumably due to compression of intracranial veins (138); and (v) BFV only serves as a reliable surrogate for CBF if the MCA diameter remains constant during the course of measurement. Since the Mx and Sx measurements do not rely on absolute BFV measurements, MCA vasospasm does not disrupt the measurement and thus, Mx, Sx and the presence of MCA vasospasm (profoundly accelerated flow velocity) can be derived from the same recordings (21). Interestingly, PRx and Mx only display a modest correlation to each other (*r* = 0.36 to 0.58), which likely reflects territorial differences (i.e., global vs. regional), measurement susceptibility to ICP and differences in model assumptions (138, 142).

Only a handful of studies have used Mx or Sx in SAH patients (Table 5) (21, 154–156). In these studies, the majority of SAH possessed elevated Mx or Sx values (21, 154, 155), although in one study, the elevated values were only found in patients who developed DCI (154). Only one study completed daily measurements over an extended time course and observed that Sx increased between days 2 and 8 in patients who develop DCI and then decline thereafter (154). Both Sx and Mx increase in the presence of vasospasm, indicating compromised autoregulation within the downstream microcirculation. In terms of prognostic value, Sx or Mx measurements in SAH patients successfully predict DCI (22, 154, 155); no studies have attempted to associate Sx or Mx values to longitudinal outcome scores in SAH patients. Taken together, these data show reasonable agreement with PRx in terms of the prevalence and time course of disrupted autoregulation and correlation to negative outcomes.

**Table 5:** Clinical studies utilizing Mean Flow Velocity Index (Mx) or Systolic Flow Velocity Index (Sx) measurements in subarachnoid hemorrhage patients.

**References**	**Citation**	**Study size**	**SAH grade**	**Critical observations**
Soehle et al., 2004	(21)	32	WFNS 1-5	Baseline Mx and Sx values in SAH patients were similar to previously reported values for healthy volunteers. Vasospasm (15/32 patients) significantly increased both Mx and Sx values.
Budohoski et al., 2012	(154)	96	WFNS 1-5	Sx values are higher in patients who develop DCI (0.09; 32/98) vs. those who do not (0.00; 66/98). Higher Sx values at days 0–5 post-ictus independently predict DCI, but not vasospasm.
Calviere et al., 2015	(155)	30	WFNS 1-3	Mx within 4 days and at 7 days post-SAH ictus, but not at 14 days post-ictus, is higher compared to previously reported values for healthy volunteers. Mx alone did not predict the development of DCI. Worsening Mx, combined with the presence of vasospasm, predicted the development of DCI.
Zweifel et al., 2010	(156)	27	WFNS 2-5	13/51 individual Mx measurements indicated disturbed autoregulation (Mx >0.15). Mx correlated with TOx measurements when both recordings time-averaged over the recording interval. Non-averaged correlations were highly variable.

*These studies included SAH patients only. DCI, delayed cerebral ischemia; Mx, Mean flow velocity index; SAH, Subarachnoid hemorrhage; Sx, Systolic flow velocity index; TOx, Tissue oxygenation index; WFNS, World Federation of Neurosurgical Societies scale*.

It should be mentioned that slow waves in blood pressure and TCD-based MCA BFV have been also analyzed with a transfer function analysis, instead of the moving Pearson correlation that generates the Mx and Sx values. Simplistically, a transfer function is Fourier decomposition of input and output signals that are transformed into sinusoids with the same frequency, but a different amplitude (gain) and a latency (shift in time; phase); a coherence function identifies conditions where estimates of gain and phase are reliable (157). When cerebral autoregulation is intact, the resistance arteries prevent/dampen the transmission of pressure fluctuations into cerebral flow (low gain and high phase). In contrast, when autoregulation is compromised, pressure fluctuations are rapidly transmitted as flow responses (high gain and low phase). Otite et al. demonstrate that higher transfer function gain and phase in SAH patients associates with angiographic vasospasm and DCI (158).

Of the remaining continuous autoregulation measures available, the ***Oxygen Reactivity*** ***Index***(correlation between slow waves in oxygen saturation and blood pressure) (114) deserves a brief mention. ORx has been assessed in SAH patients, using either an invasive oxygen probe (23, 24) or by minimally invasive near infra-red spectroscopy (NIRS) (154, 159). Budohoski et al. (154) conducted Sx measurements in parallel with ORx measures and found a similar time course for autoregulation impairment and predictive value for DCI. Oxygen reactivity measures appear to predict the development of DCI (154, 159), delayed infarction (24) and unfavorable Glasgow outcome score (GOS ≤3) (23) in SAH; however, the invasiveness and/or availability of NIRS equipment likely prohibits the widespread use of this method in a clinical setting.

### Summary of Clinical Data

Collectively, a large body of clinical data strongly support the conclusion that cerebral blood flow autoregulation is perturbed in SAH patients: this is not necessarily a surprising revelation. However, many issues hamper the overall interpretation of the literature and the utility of autoregulatory assessment as a prognostic indicator. Most notable among these issues are (i) the absence of a “gold standard” autoregulation measurement, (ii) the sparsity of studies utilizing multiple assessment techniques, and (iii) the diversity in patient populations and outcome measures. It can be argued that there is no “gold standard” method for measuring cerebral autoregulation, as every method has limitations (Table 6). Yet, the vast majority of clinical studies rely on a single approach to measure autoregulation, with ease-of-collection (or more accurately, the ability to collect data with minimal patient disturbance) moving to the forefront of practical considerations. With this in mind, it is worth acknowledging some of the key challenges and knowledge gaps with respect to clinical autoregulation measurements and its clinical use.

**Table 6:** Comparison of clinically utilized techniques to measure cerebral autoregulation in subarachnoid hemorrhage patients.

		**CBF Measurement Properties**				
**Technique**	**Invasiveness**	**Spatial resolution**	**Relative/Absolute**	**Snapshot/Continuous**	**Significant limitations**	**References**
Thermal Conductivity Probe	Invasive	Regional level	Absolute	Continuous	Measurements limited to cranial surgeries. Measurement are not completed at bedside.	(20, 117)
Carotid Artery Flow Probe	Invasive	Low/Global level	Absolute	Continuous	Measurements limited to cranial surgeries. Requires surgical implantation of flow probes.	(119)
					Measurements are not completed at bedside.	
^133^Xe Computed Tomography	Minimally or Non-Invasive	High/Local level	Absolute	Snapshot	Rapid washout limits the number of views/projections per trial. Soft tissue may attenuate signals, especially in anterior images. Method requires specialized equipment.	(101, 120, 122, 123)
					Measurements are not completed at bedside.	
Magnetic Resonance Imaging (MRI)	Minimally Invasive	High/Local level	Absolute	Snapshot	Long scan times required to obtain measurements. Very expensive equipment required.	(124)
					Measurements are not completed at bedside.	
^15^O Positron Emission Tomography	Minimally Invasive	High/Local level	Absolute	Snapshot	Long scan times required to obtain measurements. Very expensive equipment required.	(125)
					Measurements are not completed at bedside.	
Transcranial Doppler	Minimally Invasive	Regional level	Relative	Continuous	Assumes that insonified artery diameter remains constant. Not a reliable measure of CBF.	(118)
Transient Hyperemic Response Test	Minimally Invasive	Regional level	Relative	Snapshot	Highly prone to inconsistency. Assumes that MCA diameter remains constant.	(126–131)
Pressure Reactivity Index (PRx)	Invasive	Low/Global level	Relative	Continuous	ICP measurements are invasive, but may already be included in standard of care.	(46, 143, 147–151)
					Decompressive craniotomy or ventricular drains may compromise ICP measurements.	
					Measurements when ICP is high are likely unreliable	
Systolic Flow Velocity Index (Sx) or Mean Flow Velocity Index (Mx)	Non-invasive	Regional level	Relative	Continuous	Assumes that insonified artery diameter remains constant. Not a reliable measure of CBF. Requires a highly-qualified operator. Cumbersome to complete at bedside.	(21, 154–156)

*In this table, invasive techniques require surgical access, while minimally invasive techniques require injections (intravenous or intraarterial). CBF, cerebral blood flow; ICP, intracranial pressure; MCA, middle cerebral artery*.

First, static, dynamic, and continuous measurement assess different aspects and timescales of autoregulation: these may not be equally affected in pathological settings (160, 161). Comparing different methodologies, therefore, is impossible until we better define how the different assessment methods interrelate. Second, the brain has significant regional differences in vascular architecture and metabolic demand: thus, it is not surprising that regional differences in cerebral autoregulation exist in both normal (59) and pathological settings (101, 122). To add to this complexity, metabolic factors such as CO_2_ can profoundly alter autoregulation through direct actions on vascular reactivity (162): these effects would also be regional, as they should be more pronounced in ischemic regions compared to non-ischemic regions. Methodologies with limited spatial resolution will be unable to resolve regional and focal differences in autoregulation, thereby reducing their sensitivity and predictive capabilities. Third, determining the time course of autoregulatory impairment in different SAH patient populations (i.e., different severities) and more firmly establishing its relationship to DCI and other outcomes would require a significant, multi-center research undertaking; however, this information is critical for enabling accurate risk stratification that would direct clinical management. Finally, while identifying autoregulatory perturbation may indicate that an intervention is required, it does not inform the clinician with respect to *how to respond*. Thus, there is a clear need to elucidate the molecular mechanisms that perturb autoregulation in SAH. Without this knowledge, interventions will remain “blunt instruments” with some, but limited efficacy.

## Clinical Interventions for Ischemia in SAH

In the distant past (45–50 years ago and beyond), aneurysmal rebleeding was the primary cause of mortality and morbidity in SAH patients who survived the initial aneurysmal rupture. The development of aneurysm clips and effective microsurgical techniques (163, 164), pioneered in 1911 by Harvey Cushing's invention of the silver clip (165), significantly improved aneurysmal rebleeding rates. More recently, endovascular coiling has emerged as a less invasive alternative to surgical clipping: the aneurysm is accessed with a microcatheter and platinum coils are deployed into the aneurysm, stimulating the formation of a clot that permanently occludes the aneurysm (166, 167). Although re-bleeding remains a significant risk for SAH patients (11), cerebrovascular constriction and ischemia has now become the primary cause of death and disability in SAH patients who survive the initial rupture (1, 11, 12). This ischemic event is commonly termed DCI or “*delayed ischemic neurological deficit*,” due to its emergence 3–14 days post-SAH ictus (11, 12). It may also be referred to as “*symptomatic vasospasm*” or “*clinical vasospasm*,” due to its overlap with the emergence of angiographic vasospasm (13). Regardless of the term used, the clinical criteria for diagnosing DCI is “*the occurrence of focal neurological impairment (e.g., hemiparesis, aphasia, apraxia, hemianopia, or neglect), or a decrease of at least 2 points on the Glasgow Coma Scale (either on the total score or on one of its individual components [eye, motor on either side, verbal]) that lasts for at least 1 h, is not apparent immediately after aneurysm occlusion and cannot be attributed to other causes by means of clinical assessment, CT or MRI scanning of the brain, and appropriate laboratory studies*.” At present, there are only 2 interventions used to treat DCI: hyperdynamic therapy and the calcium channel antagonist nimodipine (168).

### Hyperdynamic Therapy

Hyperdynamic therapy endeavors to alleviate ischemia by increasing the CPP, sometimes in combination with altered blood rheology (169). The combined therapy is referred to as “*Triple H Therapy*,” as it includes *hypertension, hypervolemia*, and *hemodilution* as core elements (169, 170). Vasopressors, typically phenylephrine, norepinephrine or dopamine, are used to induce hypertension. Hypervolemia is induced using colloid solutions containing albumin, hexastarch, dextrans, or gelatins and serves two functions: it adds to the hypertension and induces a state of hemodilution (i.e., reduced hematocrit). There are two distinct rationales underpinning these interventions: (i) the hypertension aspect assumes that cerebral autoregulation is absent or profoundly dysfunctional in ischemic regions and thus, flow will increase in a manner proportional to CPP; and (ii) according to the Hagen–Poiseuille law, flow should increase following hemodilution, because blood viscosity decreases.

The use of hypertension for treating neurological symptoms has a long history. In 1951, Denny-Brown recognized that hypotension caused rapid neurological deterioration in patients suffering from cerebral artery disease: consequently, he proposed that raising systemic blood pressure would alleviate the cerebrovascular insufficiency and hence, the symptoms (171). Denny-Brown's postulate was not acted upon until 1967, when Farhat and Schneider used hypertension to successfully treat hemiparesis in a case series of 4 patients with cerebrovascular insufficiency (172). In some cases, a modest blood pressure elevation was sufficient to reverse ischemic symptoms (from 110/70 to 150/100 mmHg), while in other patients, rather extreme levels of hypertension were utilized (250/120 mmHg) (172). The major impediment to using hypertension in SAH patients was the significant risk of rebleeding; however, advancements in surgical methods to definitively occlude the aneurysm (i.e., clipping) opened the door for this therapeutic approach. In 1976, Kosnik and Hunt demonstrated that hypertension improved DCI symptoms in 6 of 7 surgically clipped SAH patients (173), setting the stage for what would ultimately become its routine use in SAH.

Neither Triple H therapy, nor any of its individual components, have ever been subjected to large prospective randomized clinical trials and consequently, the evidence of benefit is limited to small studies or case reports that are frequently uncontrolled and utilize different protocols. Gathier et al. planned a 240 patient randomized trial to assess the effect of hypertension on patients presenting clinical symptoms of DCI; however, the trial was prematurely halted due to slow enrollment (174). Several prior studies, however, suggested that the treatment outcomes with hypertensive or Triple H therapy are better than not intervening at all (123, 175–189). As examples: in 9 studies reporting the clinical response to hypertension in 187 SAH patients, improvement of neurological deficits ranged from 50 to 100%, with most studies reporting improvement in around 80% of patients (123, 173, 175, 176, 181–185); in 5 studies reporting long-term functional outcome at 2 to 6 months (141 patients), a good functional outcome was seen in 38 to 54% of the patients (181, 186–189). Thus, there was an impetus to incorporate the procedure into the standard of care, despite the lack of rigorous data. As documented in the 1994 and 2009 AHA guidelines for managing SAH patients (190, 191), the use of Triple-H therapy was recommended as “*a reasonable approach for treating symptomatic vasospasm*” until relatively recently. The hypervolemia component has since fallen out of favor in revised AHA therapy guidelines (168). Hypervolemia is a physiological stressor that is associated with a high incidence of adverse side effects, including: profound diuresis (176), electrolyte abnormalities (192), reflexive bradycardia (176), pulmonary edema (169, 193), dilutional coagulopathy (194), renal dysfunction (194), and cardiac failure due to fluid overload (169). Not surprisingly, some studies now indicate that positive fluid balance is associated with negative functional outcomes in SAH patients (189, 195). The revised AHA guidelines (2012) recommend (i) maintaining euvolemia and normal circulating blood volume; and (ii) inducing hypertension in patients with delayed ischemia, unless blood pressure is elevated at baseline or cardiac status precludes it (168). Interestingly, Triple H therapy, which includes the adverse effect-prone hypervolemia aspect, is still used in some clinical settings (46). The 2012 guidelines (168) remain the current standard (196), despite: (i) several recent studies challenging the notion that hyperdynamic therapy confers benefit to SAH patients suffering ischemia (197–201); (ii) systematic reviews concluding that the available evidence, which is rated “moderate-to-low grade,” does not support a recommendation (202–205); (iii) a relatively high rate of serious complications, such as cardiac arrhythmia, pulmonary edema, hemorrhagic transformation, and intracranial bleeding (181, 185); and (iv) the risk of cerebral edema developing in response to induced hypertension (39).

In the context of autoregulation, hyperdynamic therapy faces a number of significant challenges. First, as discussed previously in section 2.1, there is high inter-subject variability in the upper and lower limits of autoregulation (40); in SAH patients, this is coupled with a variable degree of perturbation (Figure 2). Hence, there is no “universal threshold” that can adequately guide clinicians with respect to effective and/or safe hypertension limits. In the absence of a reliable, real-time CBF measurement, titrating hyperdynamic therapy would require incrementally increasing pressure until neurological benefit is achieved (i.e., presumably by monitoring conscious SAH patients). Pragmatically, clinicians simply use a target pressure, with the assumption *and hope* that this will translate into improved perfusion (204).

Second, cerebral autoregulation is not uniform throughout the brain under normal conditions (59), and autoregulatory dysfunction in SAH is clearly heterogenous (101, 122). Thus, the systemic nature of hyperdynamic therapy undoubtedly elicits variable effects in different brain regions: under-perfused regions may enjoy an increase in CBF (if the hypertension is sufficient to do so), but other regions may become hyperperfused (i.e., above the upper limit of autoregulation) and prone to injury from the high pressure (206–209). These conditions, which include posterior reversible encephalopathy syndrome (PRES) and reversible leukoencephalopathy (RLS) are both causes of altered neurological function during hyperdynamic therapy that may mimic worsening DCI (206–209). This raises an interesting conundrum: since PRES and RLS are not widely reported as complications of hyperdynamic therapy, continued neurological deterioration may be interpreted as uncorrected hypoperfusion and provoke even more aggressive blood pressure augmentation, which would only further exacerbate injury. On a similar note, in cases of severe microcirculatory constriction (Figure 2A), it is possible that the only means of increasing perfusion is to breach the upper limit of autoregulation, potentially setting the stage for vasogenic edema (39). Thus, hyperdynamic therapy may benefit some SAH patients suffering from cerebral ischemia, but in many cases, the improper titration of the therapy and regional variability makes it likely that it simply substitutes another form of brain injury in the place of ischemic injury. This may partially explain why recent meta-analyses generally fail to identify improvement or harm resulting from the therapeutic intervention (202–205).

Finally, there is a presumption that the vasopressors used to induce hypertension do not appreciably alter cerebrovascular tone. Under non-pathological conditions, systemic catecholamines do not cross the blood-brain barrier and hence, increasing systemic blood pressure with norepinephrine or epinephrine does not increase CBF (210), until the upper limit of autoregulation is passed. In SAH, the blood-brain barrier is likely to be disrupted (211), permitting systemically-applied vasopressors access to the cerebrovascular smooth muscle cells, where they can then exert direct effects. This is one possible explanation for the observations by Darby et al., where certain non-ischemic regions paradoxically decreased perfusion in response hyperdynamic therapy (a potential *intracerebral steal phenomenon*) (122).

In summary, hyperdynamic therapy is a “last resort” intervention, because the underlying mechanisms causing the microcirculatory constriction are not understood. Thus, there are few options available to increase CBF when symptoms of ischemic injury occur. Hyperdynamic therapy, therefore, attempts to override the constriction with high pressure: this may increase CBF, but with risks of both systemic and central nervous system injury. The evidence supporting hyperdynamic therapy as an intervention is weak (196, 202–205) and the methods and manner of administrating the therapy are highly variable (212). Nevertheless, hyperdynamic therapy appears destined to remain a routine intervention for DCI, until a clearly superior alternative is discovered.

### Nimodipine

Nimodipine is the only FDA-approved medication for use in SAH: it belongs to the dihydropyridine (DHP) class of calcium channel blockers that inhibit L-type, voltage-sensitive calcium channels. The blockade of voltage-gated L-type calcium channels strongly impacts depolarization-stimulated vasoconstriction (213, 214), a core requirement for myogenic vasoconstriction (68). Nimodipine is rapidly absorbed from the gastrointestinal tract following oral administration and reaches peak plasma concentrations within 1 h. However, nimodipine undergoes extensive first-pass metabolism in the liver and its bioavailability following oral administration is reported to be ~13% (215, 216). Due to metabolism, nimodipine is rapidly eliminated, with an initial half-life of ~1-2 h (215, 216). Hence, nimodipine must be administered frequently (every 4 h orally) or through continuous intravenous infusion.

In order to increase cerebral perfusion, cerebrovascular resistance must decrease relative to systemic resistance. Nimodipine is characterized as a “preferential cerebrovascular dilator,” with several reports demonstrating that nimodipine stimulates an increase in cerebral blood flow at concentrations that either do not alter systemic blood pressure or elicit only modest reductions (217–220). This cerebrovascular dilatory action is preferentially exerted on small vessels (<70 μm) and there is no obvious effect on veins (220–223). Several aspects may contribute to this preferential effect:

First, nimodipine is more lipophilic than most other DHP antagonists and other calcium channel blockers, which enables it to cross the blood brain barrier: this is an obvious prerequisite for cerebrovascular efficacy. Although nimodipine crosses the blood-brain barrier, its concentration in cerebrospinal fluid (CSF) is much lower than in plasma (10-250x lower in CSF relative to plasma) (224–226). It is generally assumed, therefore, that nimodipine does not significantly bind to CSF proteins [95% of plasma nimodipine is protein-bound (215)] and is therefore absorbed by membrane structures or neuroglia within the brain. While membrane nimodipine concentrations may indeed be higher than CSF levels, the idea that nimodipine *accumulates* in cerebral cell membranes is largely not supported by available evidence: nimodipine possesses a relatively low partition coefficient, can easily wash out of membranes and has a short clinical half-life (227); radiolabeling distribution patterns show low concentrations in the brain (228). Nimodipine's cerebrovascular preference, therefore, is not likely due to a preferential distribution to the brain.

In the absence of a distribution effect (i.e., higher nimodipine concentrations in the brain), the cerebrovascular preference might entail a higher sensitivity to L-type calcium channel inhibition relative to peripheral arteries. This conclusion is primarily based on comparisons of agonist-stimulated responses (e.g., serotonin) in basilar and saphenous arteries (213, 214). However, the evidence that myogenically active cerebral resistance arteries are more sensitive to L-type calcium channel inhibition due to its ion channel composition is not clear. A systematic comparison of peripheral and cerebral artery channel expression that could more definitively address this issue is lacking.

Finally, the myogenic response is highly dependent on membrane potential, voltage-gated calcium channels, and calcium influx (68, 229, 230). Thus, vascular beds that have a high degree of autoregulation may be more susceptible to L-type calcium channel blockade, due to a more profound effect on its myogenic mechanism. Consistent with this premise, nimodipine preferentially increases both cerebral and coronary blood flow in rabbits (218): both are highly autoregulated vascular beds. Nimodipine's vasodilative effect is more pronounced in smaller arteries, which are more myogenically active than larger vessels (221, 222).

The FDA approved nimodipine for use in SAH in 1988. At the time, many of the studies supporting the use of nimodipine were “non-comparative” and relied on historical data to drive conclusions regarding the effect of nimodipine. Some notable examples of these non-comparative studies include Ljunggren et al. (231) and Auer et al. (232). While these studies generally supported the use of nimodipine therapy, their reliance on historical data as a comparator rendered them incapable of providing sound data pertaining to the overall efficacy of the nimodipine treatment. There were, however, 6 randomized placebo-controlled trials that clearly demonstrated an improvement in either neurological outcome or mortality (225, 233–237); an additional small trial that directly compared treated and untreated patients also demonstrated benefit (238) (Table 7). Long term survival assessments indicate that nimodipine reduces 3-month mortality and cerebral infarction in SAH patients (239, 243).

**Table 7:** Clinical studies involving nimodipine treatment in subarachnoid hemorrhage patients.

**References**	**Citation**	**SAH grade**	**Study size**	**Nimodipine dose**	**Placebo?**	**Primary endopoints**	**CBF measured?**	**Critical observations**
Allen et al., 1983	(225)	Not Defined	116 (56 treated)	0.35 mg/kg/4 h	YES	Neurologic, Radiographic	NO	Nimodipine reduced the incidence of severe neurological deficits, including death. Nimodipine reduced vasospasm in patients with severe outcomes, but not normal outcomes.
Philippon et al., 1986	(233)	Hunt-Hess 1-3	70 (31 treated)	60 mg/4 h	YES	Neurologic, Radiographic	NO	Nimodipine reduced neurological deficit severity when vasospasm was present. Nimodipine did not affect the incidence of neurologial deficits or vasospasm.
Petruk et al., 1988	(234)	Hunt-Hess 3-5	154 (72 treated)	90 mg/4 h	YES	Neurologic, Radiographic	NO	Nimodipine improved Glasgow Outcome Scores in Hunt-Hess 3-4 patients. Nimodipine significantly reduced neurological deficits associated with vasospasm. Nimodipine did not influence incidence or severity of vasospasm.
Mee et al., 1988	(235)	All Grades on	50 (25 treated)	60 mg/4 h	YES	Neurologic, Radiographic, CBF	**YES**	Nimodipine reduced mortality, but did not change the proportion of good/poor outcomes.
		Custom Scale						Nimodipine did not affect the incidence of vasospasm and did not change CBF.
Jan et al., 1988	(236)	Hunt-Hess 1-5	127 (73 treated)	0.03 mg/kg/h	YES	Neurologic	NO	Nimodipine improved neurological outcome in patients with vasospasm.
Pickard et al., 1989	(237)	Hunt-Hess 1-5	554 (278 treated)	60 mg/4 h	YES	Neurologic, Infarction	NO	Nimodipine reduced cerebral infarcts and poor outcomes; there was a strong tendency for reduced mortality.
Messeter et al., 1987	(238)	Hunt-Hess 1-3	20 (13 treated)	topical/i.v.	NO	Neurologic, CBF	**YES**	Nimodipine did not alter CBF, but it improved neurological outcome.
Ohman et al., 1991	(239)	Hunt-Hess 1-3	213 (109 treated)	0.03 mg/kg/h	YES	Neurologic, Infarction	NO	Nimodipine reduced mortality, but did not change the proportion of good/poor outcome.
								Nimodipine reduced the incidence of cerebral infarcts and DCI.
Rasmussen et al., 1999	(240)	Hunt-Hess 3-5	8 (pre/post)	0.03 mg/kg/h	NO	CBF, autoregulation, CRMO_2_	**YES**	Nimodipine did not alter CBF or autoregulation. Nimodipine may improve CRMO_2_ during hypotension.
Choi et al., 2012	(241)	Hunt-Hess 3-5	16	30–60 mg/4 h	NO	MAP, CBF	**YES**	Each nimodipine dose caused small decreases in MAP and CBF.
Hänggi et al., 2008	(242)	WFNS 1-4	26 (pre/post)	Intra-arterial	NO	CBF, radiographic	**YES**	In patients with severe vasospasm refractory to systemic nimodipine, intra-arterial nimodipine transiently reduced vasospasm and increased perfusion.

*These studies included SAH patients only. CBF, Cerebral blood flow; CRMO_2_, cerebral metabolic rate of oxygen; DCI, delayed cerebral ischemia; MAP, mean arterial pressure; SAH, Subarachnoid hemorrhage; WFNS, World Federation of Neurosurgical Societies scale*.

Since nimodipine possesses vasodilatory properties, a vascular mechanism was presumed. However, there is scant evidence for an effect on cerebral perfusion in SAH patients. Only 2 of the controlled trials (1 placebo controlled) that preceded FDA approval conducted CBF measurements: neither found evidence that nimodipine increased cerebral perfusion (235, 238). In 4 studies, angiographic vasospasm was assessed: in 1 trial, nimodipine reduced the severity of angiographic vasospasm in patients with severe neurological outcomes (225); however, the remaining trials showed no difference in the incidence or severity of angiographic vasospasm (233–235). In summary, the FDA approved nimodipine based on solid evidence that it improves neurological outcomes and mortality after SAH across all grades, except perhaps for the most severe cases (244); however, there is minimal-to-no evidence that nimodipine improves cerebral perfusion as its mechanism of action (Table 7). It is also notable that nimodipine is not an effective treatment for acute ischemic stroke (245) or traumatic brain injury (246), both of which involve ischemic injury as a component of the pathologies.

Only a handful of studies have examined the effect of nimodipine on cerebral autoregulation in humans and animal models (240, 247–249): the majority of these studies found that nimodipine has no effect on CBF autoregulation; in one experimental study, nimodipine improved cerebral autoregulation in rats following SAH (measured by ^133^Xe clearance) (250). For obvious reasons, nimodipine has not been rigorously trialed against a placebo in SAH patients to determine its mechanism of action. While there is some evidence that nimodipine treatment increases cerebral perfusion in healthy volunteers (219, 247), only a handful of studies have attempted to determine whether nimodipine increases cerebral perfusion in SAH patients (235, 238, 240–242): these trials are difficult to interpret, due to the lack of an untreated control. Of the trials that use reasonably reliable CBF measurement methods (e.g., ^133^Xe clearance), the results are generally unsupportive of a perfusion effect (Table 7). Rasmussen et al. measured CBF in patients with severe SAH prior to and following nimodipine treatment and observed no effect on CBF or cerebral autoregulation (240). Choi et al. measured CBF following successive nimodipine dosing and generally found marginal reductions in CBF with each dose, concomitant with reductions in MAP (241). Hänggi et al. demonstrated that intra-arterial administration of nimodipine improved perfusion in patients with refractory vasospasm, but only transiently (242).

The absence of a clear vascular effect has spurred the search for non-vascular mechanisms of action that could explain nimodipine's beneficial effect in SAH. The attenuation of cortical spreading depolarizations is one potential non-vascular mechanism that has received significant attention (251). Spreading depolarizations are slow propagating waves of neuronal and glial mass depolarization that can occur when brain tissue is exposed to noxious stimuli following neurological injury (251). Under normal conditions, the depolarization is associated with a contaminant increase in regional CBF, to match the increased metabolic demand. However, after SAH and ischemic stroke, the oligemic response is converted to a ischemic response, resulting in paradoxical vasoconstriction and spreading ischemia. Since neurons express high levels of L-type calcium channels (252), nimodipine could directly protect neurons by inhibiting these spontaneous depolarizations. In support of this potential mechanism of action, spreading depolarizations are known to occur in SAH patients and are associated with delayed ischemic injury (253, 254); subarachnoid blood can acutely trigger spreading depolarizations that cause infarctions (255); and nimodipine is capable of attenuating spreading depolarizations (256, 257). Nimodipine can directly protect neurons from ischemic damage by this mechanism (258, 259) and could prevent electrical changes in response to oxygen/glucose deprivation (260). Taken together, there is a reasonable basis to propose that direct neuroprotection explains nimodipine's positive effect in SAH.

While a neuroprotective mechanism can confer benefit during ischemic episodes in SAH, directly targeting the perfusion deficit (i.e., the original intent of the treatment) would undoubtedly be more effective. However, globally targeting the cerebral microcirculation with a vasodilator has many of the same shortcomings as hyperdynamic therapy. Specifically, there is both normal or pathological heterogeneity in autoregulatory status within the cerebral microcirculation: thus, global treatment could undermine autoregulation in regions where it functions properly, potentially leading to hyperperfusion, edema and/or potentially, a *steal phenomenon*. It should also be noted that since the myogenic response, the basis of autoregulation, depends on L-type calcium channels (68, 229, 230), careful titration would be required to maintain autoregulatory function. As an illustration, Hockel et al. delivered nimodipine as a continuous intra-arterial infusion *via* the carotid artery, in order to maximally deliver a high nimodipine dose to the cerebral microcirculation (261). The authors measured an increase in PRx and oxygen tension, suggesting a vascular effect, albeit the overall effect was transient (6 h) (261). The increase in PRx value indicates deteriorated autoregulation, which should not occur unless the treatment compromises autoregulation as a whole (i.e., the myogenic mechanism may be ablated in some regions).

In summary, it appears unlikely that orally administered nimodipine elicits significant and sustained cerebrovascular dilation and increased perfusion. As a consequence, a neuroprotective mechanism is now favored. While it would be preferable to target the vascular mechanism, the global use of vasodilators is problematic and there are currently no alternative approaches available.

## Discussion

Cerebral autoregulation is a crucial homeostatic mechanism: its disruption in SAH can profoundly compromise cerebral perfusion and consequently, cause ischemic injury. Autoregulatory status can be measured in a clinical setting: while these measures are generally predictive of outcome, future efforts need to establish a “gold standard” procedure with sufficient numbers in order provide appropriate diagnostic and prognostic guidance. However, the absence of interventions that can correct perturbed autoregulation is a dire problem: a substantial research investment is required on this front, as the number of interventional options at the clinician's disposal is limited.

As reviewed by Daou et al., an array of pharmacological interventions have been tested for efficacy in SAH (262). Many of these interventions displayed early promise in reducing ischemic injury in SAH, only to later fail in larger trials: some notable examples include clazosentan (endothelin-1 receptor blocker) (15–18), nicardipine (263), magnesium (voltage-dependent calcium channel blocker) (264, 265), statins (pleiotropic vascular and neuroprotective effects) (266, 267), tirilizad (antioxidant) (268), and erythropoietin (pleiotropic vascular and neuroprotective effects) (269, 270). Given that the mechanisms underlying SAH-induced microvascular dysfunction have not been adequately defined, these interventions were predicated on their known vascular effects and/or their successful use in other pathological settings. However, since the etiology of microvascular dysfunction in SAH is largely uncharacterized and probably multifactorial, the failure to properly target a causal mechanism undoubtedly contributed to these interventions' lack of efficacy.

There are few studies that systematically investigate how myogenic reactivity, the underlying basis of autoregulation, changes in SAH (87, 93, 95, 96, 98–100). Isolating and cannulating small resistance arteries that are 0.1 mm in diameter and 0.5–0.8 mm in length is time consuming and technically demanding work; it is difficult to conduct biochemical assessments on artery samples, as the tissue amounts are highly limited; and molecular manipulations are generally limited to testing arteries from gene deletion models. As a consequence, only a small number of researchers utilize pressure myography as a core research technique and from there, only a small proportion of these researchers study SAH, as it is considered an orphan disease. In the current research climate, where high-throughput projects are favored for funding, it is hard to attract new researchers into this challenging discipline, despite the desperate need.

**Figure 3:**
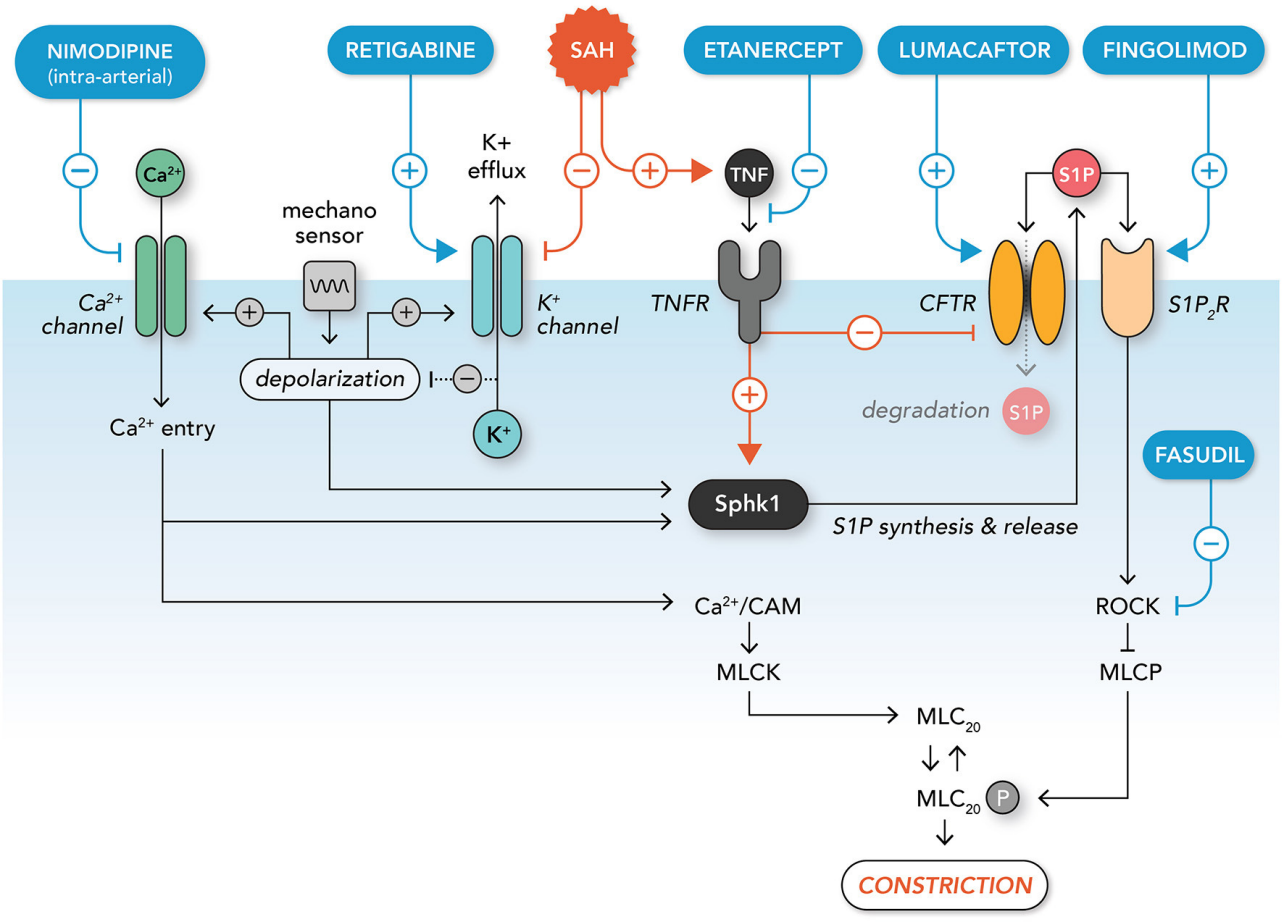
Molecular mechanisms augmenting microvascular constriction in subarachnoid hemorrhage and potential interventions. A mechanosensitive complex initiates myogenic vasoconstriction *via* membrane potential depolarization: this leads to calcium entry *via* opening of voltage-gated calcium channels; voltage gated potassium channels also open, leading to hyperpolarizing potassium efflux that limits the extent of depolarization (negative feedback). Intracellular calcium activates calmodulin (CAM), myosin light chain kinase (MLCK) and the contraction apparatus. In addition, calcium entry and depolarization lead to the activation of sphingosine kinase 1 (Sphk1), which synthesizes and releases sphingosine-1-phosphate (S1P) into the extracellular compartment. Extracellular S1P activates the S1P2 receptor subtype (S1P_2_R), thereby activating the Rho-associated protein kinase (ROCK) signaling pathway. ROCK signaling inhibits myosin light chain phosphatase (MLCP), which enhances MLCK's activation of the contractile apparatus. Extracellular S1P is sequestered from S1P_2_R by the cystic fibrosis transmembrane conductance regulator (CFTR), which transports S1P across the plasma membrane for degradation. In subarachnoid hemorrhage (SAH), potassium channels are down-regulated, leading to enhanced depolarization through disinhibition. SAH also stimulates inflammatory tumor necrosis factor signaling, which down-regulates CFTR expression and enhances the activation of Sphk1. This increases pro-constrictive S1P / S1P_2_R / ROCK signaling, which enhances constriction *via* the inhibition of MLCP. Therapeutics targeting these pathological processes include: (i) intra-arterially-delivered nimodipine (calcium channel blocker; limits calcium entry), (ii) retigabine (potassium channel activator; reduces depolarization), (iii) etanercept (inhibits TNF signaling; reduces S1P synthesis and increases S1P degradation), (iv) lumacaftor (increases CFTR expression; increases S1P degradation), (v) fingolimod (stimulates S1P receptor internalization; inhibits ROCK activation), and (vi) fasudil (ROCK inhibitor; limits inhibition of MLCP).

Based on the available myogenic reactivity studies utilizing SAH models, several potential therapeutic targets have been proposed (Figure 3), including Kv channels, tumor necrosis factor (TNF), S1P receptor subtype 2 (S1P_2_R), Rho-associated protein kinase (ROCK) and the cystic fibrosis transmembrane conductance regulator (CFTR). It is worth considering how these might fare as interventions (Table 8).

**Table 8:** Potential interventions for treating dysfunctional autoregulation in subarachnoid hemorrhage.

**Molecular target**	**Target function**	**Pathological mechanism**	**Intervention**	**Mechanism of action and potential negative effects on autoregulation**
Potassium channels (Kv and BK)	Counteracts depolarization with a hyperpolarizing current	Channels are downregulated in SAH resulting in enhanced depolarization	Retigabine Zonisamide	Intervention activates potassium channels, thereby reducing cellular excitability. Overdosing potentially abolishes myogenic reactivity, thereby eliminating autoregulation. Intervention is not specific to myogenic reactivity or autoregulation.
Calcium channels (L-Type)	Permits extracellular calcium entry, which activates molecular effectors of cellular contraction.	Calcium influx is augmented in SAH due to enhanced depolarization	Nimodipine	Intervention attenuates depolarization-dependent calcium influx. Overdosing potentially abolishes myogenic reactivity, thereby eliminating autoregulation. Intervention is not specific to myogenic reactivity or autoregulation
S1P_2_R	Enhances calcium sensitivity *via* ROCK-dependent inhibition of MLCP	Calcium sensitivity is augmented in SAH *via* enhanced S1P signaling	Fingolimod	Intervention reduces calcium sensitivity by antagonizing S1P_2_R signaling. Strong S1P_2_R antagonism may abolish myogenic reactivity, thereby eliminating autoregulation. Intervention is immunosuppressive.
ROCK	Enhances calcium sensitivity *via* MLCP inhibition	Calcium sensitivity is augmented in SAH *via* enhanced S1P signaling	Fasudil	Intervention reduces calcium sensitivity by inhibiting ROCK. Strong ROCK inhibition may abolish myogenic reactivity, thereby eliminating autoregulation.
TNF	Pathological mechanism only. Stimulates S1P production and downregulates CFTR expression.	Enhanced S1P signaling augments calcium influx and calcium sensitivity	Etanercept	Intervention normalizes myogenic reactivity and autoregulation by eliminating the pathological enhancement of S1P signaling. Intervention is immunosuppressive.
CFTR	Antagonizes S1P_2_R signaling by sequestering S1P away from receptors	Calcium sensitivity is augmented in SAH *via* enhanced S1P signaling	Lumacaftor	Intervention normalizes myogenic reactivity and autoregulation by eliminating the pathological enhancement of S1P_2_R signalling.

*BK, calcium activated large conductance potassium channels; CFTR, Cystic fibrosis transmembrane conductance regulator; Kv, Voltage-gated potassium channel; MLCP, myosin light chain phosphatase; ROCK, Rho-associated protein kinase; SAH, subarachnoid hemorrhage; S1P, Sphingosine-1-phosphate; S1P_2_R, Sphingosine-1-phosphate receptor 2; TNF, Tumor necrosis factor*.

Several studies using isolated cerebral arteries (98) and/or primary smooth muscle cells derived from cerebral arteries (96, 98, 271) demonstrate that SAH reduces potassium currents, leading to enhanced depolarization and calcium entry in response to pressure (96, 100, 271). These results are buttressed by experiments demonstrating that oxyhemoglobin reduces potassium channel expression *in vitro* (272–274). The specific potassium channel types/subtypes affected have not been fully defined and may involve K_v_1.5, K_v_2.1, K_v_2.2, K_v_7, or BK channels (100, 271, 275, 276). Both K_v_ channel activators (e.g., retigabine) (276) and BK channel activators (e.g., zonisamide) (277, 278) are clinically available and have been used in experimental SAH settings; however, to our knowledge, these therapeutics have not been utilized to clinically treat SAH. Both retigabine and zonisamide are typically used as anti-seizure medications and are associated with several adverse central nervous system effects, including somnolence, dizziness and confusion (279, 280). Further, Kv and BK channels are expressed in neurons and many other excitable cell types and thus, widespread adverse effects could occur. These issues probably preclude their use clinical use in SAH.

The remaining targets (TNF, S1P, S1P_2_R, CFTR, and ROCK) may all be functionally linked into a pathological signaling chain (75). Briefly, our previous work shows that soluble TNF [i.e., TNF released *via* shedding (281–283)] enhances S1P signaling in cerebral arteries following SAH, by augmenting S1P synthesis and preventing S1P degradation (87, 93). Specifically, TNF enhances S1P synthesis *via* sphingosine kinase 1 activation (284); simultaneously, it reduces S1P degradation, *via* the down-regulation of CFTR protein expression (87, 93), a critical S1P transporter (285, 286) that sequesters S1P from its receptors (287). In cerebral vessels, S1P significantly enhances vascular tone and myogenic vasoconstriction (87, 285, 288–291), primarily *via* the S1P_2_R subtype (87). Mechanistically, S1P_2_R activation enhances calcium sensitivity (i.e., yielding more constriction for a given increase in intracellular calcium) *via* the activation of the ROCK signaling cascade (292).

There are several lines of evidence supporting TNF's role in SAH: (i) cerebrospinal TNF levels rise and peak 4–10 days post-SAH ictus (293–295), the timeframe when delayed autoregulatory disruption and ischemia generally occurs; (ii) TNF levels associate with perturbed cerebral flow velocities and poor outcomes (293, 296, 297); and (iii) anti-TNF therapy (i.e., etanercept or adalimumab) reduces vascular constriction and/or neurological injury in experimental SAH (298–300). Likewise, cerebrospinal S1P levels are also substantially elevated in SAH patients (301) and attenuating S1P receptor activity, either with an S1P_2_R antagonist (JTE-013) (87) or fingolimod (stimulates S1P receptor internalization) (302, 303), improves perfusion and outcomes in experimental SAH. The ROCK inhibitor fasudil, which inhibits the downstream signals associated with S1P_2_R activation, is currently used prophylactically in Japan and China, with some clinical studies demonstrating evidence of benefit (304–306). Collectively, these data strongly support that SAH activates a pro-constrictive TNF/S1P signaling axis. It should be noted, however, that there is limited clinical trial data from which to draw from and the purported benefits of fasudil remain to be confirmed in large randomized controlled clinical trials (307).

There are no clinically approved S1P_2_R antagonists. Fingolimod is approved to treat multiple sclerosis (308) and has been clinically tested in small trials involving acute cerebral stroke (309) and intracerebral hemorrhage (310). While these stroke trials did not observe adverse effects associated with treatment (309, 310), fingolimod is known to be immunosuppressive and is associated with several adverse effects, including infections, elevated liver enzymes, central nervous system effects [e.g., headache, dizziness and in some cases, leukoencephalopathy (311)] and cardiac effects (bradycardia and atrioventricular block) (308). The immunosuppression and known adverse effects have likely deterred fingolimod's use in SAH clinical trials. Like fingolimod, TNF antagonists (e.g., etanercept, adalimumab) are also immunosuppressive and bear a high risk of permitting opportunistic infections (312); additionally, the large physical size of the therapeutics (~150 kDa) would necessitate a compromised blood-brain barrier or intrathecal delivery in order to reach its targets within the brain. The immunosuppressive effects and risk of secondary infection probably deter the use of anti-TNF therapeutics. Fasudil appears to have reasonable safety in SAH patients (313). One possible drawback to fasudil treatment is that it likely requires proper titration, as strong ROCK inhibition would compromise myogenic reactivity altogether (90); fasudil may also compromise myogenic responses in cerebral vessels that are not perturbed by SAH. As stated previously, fasudil's efficacy has not been conclusively ascertained (307), as no large, randomized controlled clinical studies have been undertaken.

This narrows the already short list of candidates to one intriguing entity: CFTR therapeutics (75). Experimentally, the CFTR “corrector” therapeutic lumacaftor (314) increases wild-type CFTR expression in cerebral arteries by a proteostatic mechanism (93, 315): this modification normalizes the augmented myogenic reactivity in cerebral arteries derived from mice with experimental SAH, but has no apparent effect in control/sham arteries (93). Not surprisingly, lumacaftor successfully increases cerebral perfusion and protects against neuronal injury in experimental SAH (93). Additionally, skeletal muscle resistance arteries do not appear to appreciably express CFTR (93, 316): since these peripheral arteries would not be affected by the CFTR therapeutics, the chances of developing hypotension in response to treatment appears to be limited. Collectively, this represents the quintessential “magic bullet” scenario, where the therapeutic specifically targets a pathological cerebrovascular mechanism.

CFTR therapeutics are generally considered safe and are well-tolerated, although this pertains to its use in cystic fibrosis patients. There is limited safety data available for non-cystic fibrosis patients; however, CFTR therapeutics have been shown to be well-tolerated in a small cohort of healthy volunteers (317) and smokers with chronic obstructive pulmonary disease (318). There are two important caveats to the use of CFTR therapeutics in SAH patients: first and foremost, there is a large translational gap between animals and human subjects and it remains to be determined whether CFTR is a regulator of cerebrovascular tone in humans. On this note, our previous translational work comparing human/mouse mesenteric and skeletal muscle artery CFTR expression and function displayed excellent comparability (316), providing reasonable grounds to predict that human cerebral arteries will be modulated by CFTR therapeutics. Nevertheless, proof-of-principle clinical trials are needed. The second caveat pertains to the cost of the medication. Because cystic fibrosis is a rare disease, the medications are very expensive: this is necessary in order for the drug companies to recoup the heavy costs invested into their clinical trials. Since SAH is itself a rare disease, short-term use (i.e., a week to a few months) may incur a tolerable expense; it will be much more cost effective when the medications can be repurposed after they are off-patent.

## Conclusion

Reliably improving cerebral perfusion in SAH patients has proven challenging: while many pathological mechanisms have been proposed, understanding why vascular reactivity changes in SAH remains a serious knowledge deficiency. Consequently, there have been no therapeutic interventions developed within the last 35 years that conclusively ameliorate ischemia in SAH, with the notable exception of nimodipine (which probably works by a different mechanism than intended). Myogenic reactivity and its systemic counterpart, cerebral autoregulation, are crucial homeostatic mechanisms that are perturbed in SAH. Understanding the molecular basis for this microvascular dysfunction, coupled with advances in clinical monitoring of autoregulation, will provide the best prospect of identifying new interventions that successfully restore autoregulation and perfusion in SAH patients.

## Author Contributions

DL wrote the first draft of this article. All authors contributed equally to the conceptualization, literature research, and revision of the final manuscript.

## Conflict of Interest

DL is a consultant for Qanatpharma AG (Stans, Switzerland). SS-B is executive board member of Qanatpharma AG and Aphaia Pharma AG (Zug, Switzerland). Neither Qanatpharma AG nor Aphaia Pharma AG had any financial or intellectual involvement in this article. The remaining author declares that the research was conducted in the absence of any commercial or financial relationships that could be construed as a potential conflict of interest.

## Publisher's Note

All claims expressed in this article are solely those of the authors and do not necessarily represent those of their affiliated organizations, or those of the publisher, the editors and the reviewers. Any product that may be evaluated in this article, or claim that may be made by its manufacturer, is not guaranteed or endorsed by the publisher.
